# Genetics and Beyond – The Transcriptome of Human Monocytes and Disease Susceptibility

**DOI:** 10.1371/journal.pone.0010693

**Published:** 2010-05-18

**Authors:** Tanja Zeller, Philipp Wild, Silke Szymczak, Maxime Rotival, Arne Schillert, Raphaele Castagne, Seraya Maouche, Marine Germain, Karl Lackner, Heidi Rossmann, Medea Eleftheriadis, Christoph R. Sinning, Renate B. Schnabel, Edith Lubos, Detlev Mennerich, Werner Rust, Claire Perret, Carole Proust, Viviane Nicaud, Joseph Loscalzo, Norbert Hübner, David Tregouet, Thomas Münzel, Andreas Ziegler, Laurence Tiret, Stefan Blankenberg, François Cambien

**Affiliations:** 1 Medizinische Klinik und Poliklinik, Johannes-Gutenberg Universität Mainz, Mainz, Germany; 2 Institut für Medizinische Biometrie und Statistik, Universität zu Lübeck, Universitätsklinikum Schleswig-Holstein, Lübeck, Germany; 3 INSERM UMRS 937, Pierre and Marie Curie University and Medical School, Paris, France; 4 Institut für Klinische Chemie und Laboratoriumsmediizin, Johannes-Gutenberg Universität Mainz, Mainz, Germany; 5 Boehringer Ingelheim Pharma GmbH and Co. KG, Biberach, Germany; 6 Department of Medicine, Brigham and Women's Hospital, Boston, Massachusetts, United States of America; 7 Max Delbrück Center for Molecular Medicine, Berlin, Germany; VU University Medical Center and Center for Neurogenomics and Cognitive Research, VU University, Netherlands

## Abstract

**Background:**

Variability of gene expression in human may link gene sequence variability and phenotypes; however, non-genetic variations, alone or in combination with genetics, may also influence expression traits and have a critical role in physiological and disease processes.

**Methodology/Principal Findings:**

To get better insight into the overall variability of gene expression, we assessed the transcriptome of circulating monocytes, a key cell involved in immunity-related diseases and atherosclerosis, in 1,490 unrelated individuals and investigated its association with >675,000 SNPs and 10 common cardiovascular risk factors. Out of 12,808 expressed genes, 2,745 expression quantitative trait loci were detected (*P*<5.78×10^−12^), most of them (90%) being *cis*-modulated. Extensive analyses showed that associations identified by genome-wide association studies of lipids, body mass index or blood pressure were rarely compatible with a mediation by monocyte expression level at the locus. At a study-wide level (*P*<3.9×10^−7^), 1,662 expression traits (13.0%) were significantly associated with at least one risk factor. Genome-wide interaction analyses suggested that genetic variability and risk factors mostly acted additively on gene expression. Because of the structure of correlation among expression traits, the variability of risk factors could be characterized by a limited set of independent gene expressions which may have biological and clinical relevance. For example expression traits associated with cigarette smoking were more strongly associated with carotid atherosclerosis than smoking itself.

**Conclusions/Significance:**

This study demonstrates that the monocyte transcriptome is a potent integrator of genetic and non-genetic influences of relevance for disease pathophysiology and risk assessment.

## Introduction

The transcriptome, i.e. the whole set of RNA transcripts in a cell, is generally conceived as a system whose major function is to pass information encoded in the genome sequence to the realm of phenotypes that underlie physiological and pathological traits. This messenger paradigm justifies the current interest for the genetics of gene expression [1]–[6] which has been further enhanced by the numerous associations between genetic markers and diseases reported in recent genome-wide association studies (GWAS) and the expected relevance of genome wide expression (GWE) to characterize the biological basis of these associations [7]–[10]. However the variability of gene expression not only reflects genetic variation but depends on other factors as well such as environmental exposures [11], [12], metabolic conditions [13], ageing [14], [15] or gender [16]–[17].

Based on these premises we reasoned that if the state of the transcriptome and its changes are important determinants of cell functions, differences in transcript abundance whatever their origin, genetic or non genetic, may contribute to disease pathogenesis. Moreover, the transcriptome might integrate information from numerous sources and inform on the current pathophysiological state of the organism. To assess these possibilities, a global characterization of the variability of the transcriptome, integrating genetic and non genetic influences was undertaken. The study focused on peripheral blood monocytes because these cells may be isolated from an easily accessible tissue and play a key role in the pathogenesis of immune disorders and atherosclerosis-related diseases [18]. In addition, working on a single cell type reduces the complexity of transcriptome data and may avoid possible biases resulting from the heterogeneous cell-types distribution in different samples as it is the case when using whole blood or leucocytes RNAs.

## Results

### The genome-wide expression of circulating monocytes

To reduce potential artefacts, fresh samples were collected and processed in a short period of time according to a very strict protocol. Monocytes were obtained from 1,490 unrelated individuals, 730 women and 760 men, aged 35 to 74 years, recruited in the Gutenberg Heart Study (GHS), a community-based project conducted in a single centre in the region of Mainz (Germany) (Table 1). GWE profiles were generated using *Illumina* Human HT-12 expression BeadChips, and after normalization and filtering out genes undetected in monocytes or non-well characterized (see Materials and Methods), 12,808 expressions traits (averaged over probes) remained for analysis.

**Table 1:** Description of the GHS study population.

	Men	Women	*P*-value
N	760	730	
Age (years)	56.4 (10.6)	53.9 (11.2)	2.4×10^−5^
BMI (kg/m^2^)	27.6 (3.9)	26.2 (5.1)	1.2×10^−8^
HDL cholesterol (mg/dL)	54.4 (14.9)	69.2 (17.8)	2.2×10^−16^
LDL cholesterol (mg/dL)	133.7 (36.1)	133.0 (36.8)	NS
Triglycerides (mg/dl)	143.2 (97.5)	114.4 (56.8)	2.1×10^−12^
Systolic blood pressure (mmHg)	135.8 (16.7)	128.5 (18.2)	2.3×10^−16^
Diastolic blood pressure (mmHg)	85.2 (9.6)	81.2 (9.5)	5.4×10^−16^
Current smoker	128 (16.8%)	113 (15.5%)	NS
Plasma CRP (mg/L) (sqrt)	1.509 (0.818)	1.545 (0.743)	NS
Plasma glucose (mg/dL)	97.8 (18.5)	91.8 (15.4)	1.1×10^−4^

*Values are means (SD) or numbers (%).*

### Identification of eSNPs and eQTLs

Genotyping was performed using *Affymetrix* 6.0 arrays. After filtering out SNPs poorly performing or having a minor allele frequency <0.01, 675,350 SNPs were kept for further analyses. All associations between SNPs and expression traits with a *P-*value <10^−5^ (*n*>225,000) were stored in the “GHS_Express” database (“GHS_Express” is available online, see Methods S1). At a study-wise threshold of significance correcting for the number of SNPs and expressions (*P*<5.78×10^−12^), 37,403 associations, involving 29,912 SNPs and 2,745 expression traits (referred to as eSNPs and eQTLs, respectively), were identified (Table 2). The median number of eSNPs by eQTL was 11 with an interquartile range of 4 to 26. Owing to its large sample size, the study had an 80% power to detect a SNP effect accounting for 4% or more of the variability (R^2^) of any expression trait. Among the 2,745 significant eQTLs, the R^2^ observed for the best eSNP varied from 3.1% to >80% with a median of 7.7%. For 290 eQTLs, the R^2^ was greater than 25%.

**Table 2:** Number of gene expression-by-SNP associations at various levels of significance.

Significance level	Minimum R^2^ $	Total number of associations	*cis*/*trans* ratio for associations	Total number of associated expressions (eQTLs)	*cis/trans* ratio for eQTLs	Total number of associated SNPs (eSNPs)	*cis/trans* ratio for eSNPs
<10^−6^	0.016	93491	2.1	8575	0.5	67190	2.4
<10^−8^	0.022	54749	7.3	3857	3.0	41425	11.2
<10^−10^	0.028	42421	9.8	2998	6.0	33339	16.3
**<5.78×10^−12^**	**0.031**	**37403**	**10.7**	**2745**	**7.1**	**29912**	**17.1**
<10^−15^	0.042	27330	12.7	2180	9.5	22591	17.8
<10^−20^	0.057	19655	14.7	1725	12.8	16883	19.2
<10^−25^	0.071	15015	16.4	1429	16.2	13045	21.5
<10^−35^	0.099	9673	17.1	1031	21.6	8516	22.9
<10^−50^	0.140	5873	14.0	712	28.8	5224	21.7
<10^−100^	0.263	1790	10.5	290	28.1	1598	11.1
<10^−150^	0.371	922	5.5	156	21.4	772	5.9
<10^−200^	0.463	635	3.7	97	15.3	504	3.9
<10^−300^	0.606	321	1.7	38	11.7	213	1.7

$ *Minimum R^2^ (proportion of gene expression variability explained by a SNP) observed for a given significance level. Numbers corresponding to study-wise significance are shown in bold. For investigating cis associations or performing any other hypothesis-based test, lower levels of significance may be considered.*

### Cis versus trans associations

**Figure 1:**
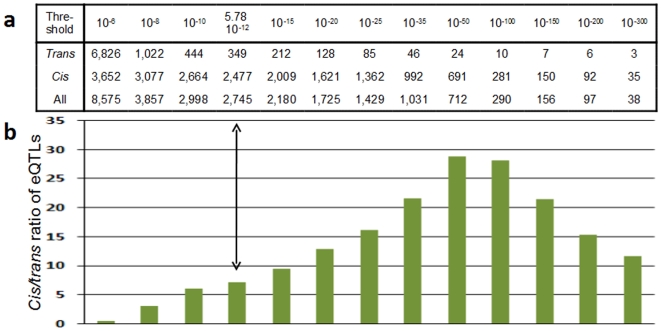
Number of eQTLs according to the significance threshold adopted and corresponding *cis/trans* eQTL ratio. The vertical arrow indicates the study-wise level of significance correcting for the number of hypotheses tested. Some eQTLs being associated with both *cis* and *trans*-acting eSNPs, the sum of *cis* and *trans* eQTLs is greater than the total number of eQTLs.

Associations involving SNPs located within 1 Mb of either the 5′ or 3′ end of the associated gene were considered *cis* (File S1) and other associations were considered *trans*. In accordance with previous results [2]–[6], most of the genetic variability affecting the transcriptome was of *cis* origin. At study-wise significance, the number of *cis* and *trans* eQTLs were 2,477 and 349, respectively, yielding a *cis*/*trans* ratio of 7.1 (81 eQTLs were both *cis-* and *trans*-modulated). At less stringent levels of significance the number of *trans* associations considerably increased, as expected by chance, whereas the number of *cis* eQTLs only modestly increased, indicating that the high stringency used for *cis* eQTLs identification did not result in an important under-estimation of the true number of *cis* eQTLs (Figure 1).

### Comparison with previous GWAS of gene expression

**Figure 2:**
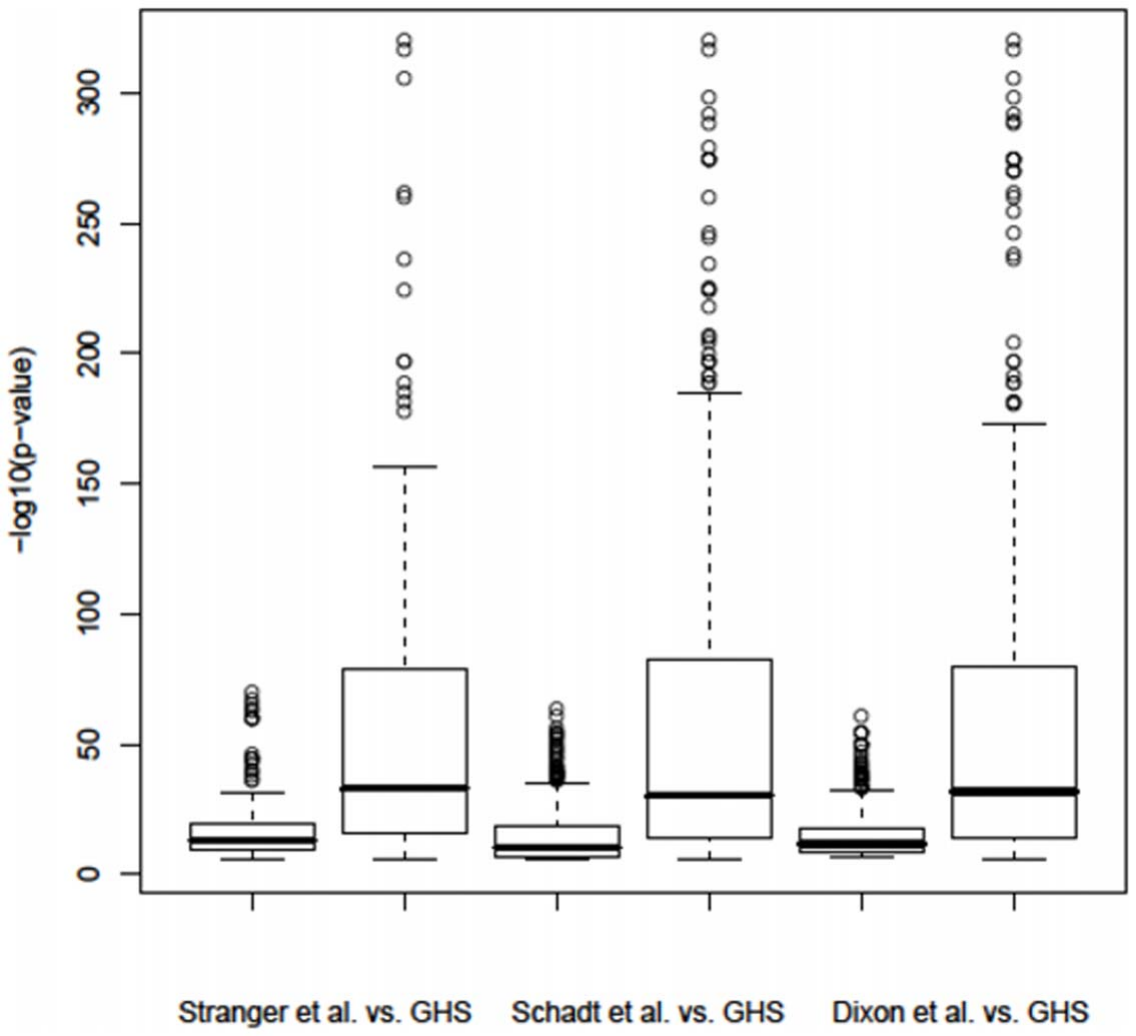
Comparison of the distributions of *P*-values of *cis* eQTLs reported as significant in three previous association studies with *P*-values observed in GHS for the same eQTLs. For each of the 3 comparisons, we selected in GHS the subset of gene expressions claimed as significant in the study of comparison. Only autosomal genes were considered in these comparisons. The data used to generate this figure are provided in Files S2–S4. See also footnote of Table S3 for details.

We examined the overlap between the *cis* eQTLs identified in the present study and those found in three previous association studies in which gene expression was explored in LCLs [1], [2] and hepatic cells [3]. For this comparison, a significance threshold of 3.9×10^−6^ (Bonferroni-corrected for 12,808 genes) was used for the analysis of eQTLs in GHS data, corresponding to a single hypothesis tested per gene. Among the *cis* eQTLs considered significant in each of the studies and involving expression traits detected in GHS, 66.7%, 56.5% and 54.1%, respectively, were significant in our data (Table 3 , Files S2–S4). The proportion of *cis* eQTLs that replicated in GHS increased with the increasing level of significance reported in each study, consistent with the fact that stronger associations are more robust and more likely to be shared by different types of cells. These comparisons revealed a relatively high rate of replication of the previous findings in GHS. However, as a consequence of its greater power, *P*-values observed in GHS were considerably lower than those previously reported (Figure 2).

**Table 3:** Number of *cis* eQTLs identified in previous studies and replicated in GHS.

	Stranger et al.		Dixon et al.		Schadt et al.	
Level of signifi-cance	Number of eQTLs at level of significance	Percent signifi-cant in GHS*	Number of eQTLs at level of significance	Percent significant in GHS*	Number of eQTLs at level of significance	Percent signifi-cant in GHS*
>10^−8^	86	55.8	110	50.9	928	47.9
10^−8^–10^−10^	63	69.8	162	50.0	168	57.7
10^−10^–10^−15^	144	63.2	237	54.8	211	57.3
10^−15^–10^−20^	60	70.0	102	60.7	120	66.7
10^−20^–10^−25^	38	89.5	73	65.7	73	67.1
≤10^−25^	48	70.8	89	67.4	103	73.8
All	439	66.7	773	56.5	1603	54.1

* *Comparisons were based on sets of gene expressions overlapping between each study and GHS and were restricted to autosomal cis eQTLs. All cis eQTLs considered significant in each study were retrieved and replication was assessed in GHS (P<3.9×10^−6^ correcting for 12,808 gene expressions).*

*For Stranger et al* [1], *data were extracted from Table S2. We considered as significant the associations found in at least 3 HAPMAP populations. For Dixon et al* [2], *data were extracted from Table S1 and trans eQTLs were excluded. Matching of probes was done using a table provided by the authors on their web site. For Schadt et al* [3], *cis eQTLs considered significant (First.Pass.Indicator set to 1) were extracted from Table S3. For each eQTL, we selected in GHS the P-value of the best cis eSNP. The full data used to generate this table are provided in Files S2–S4.*

**Figure 3:**
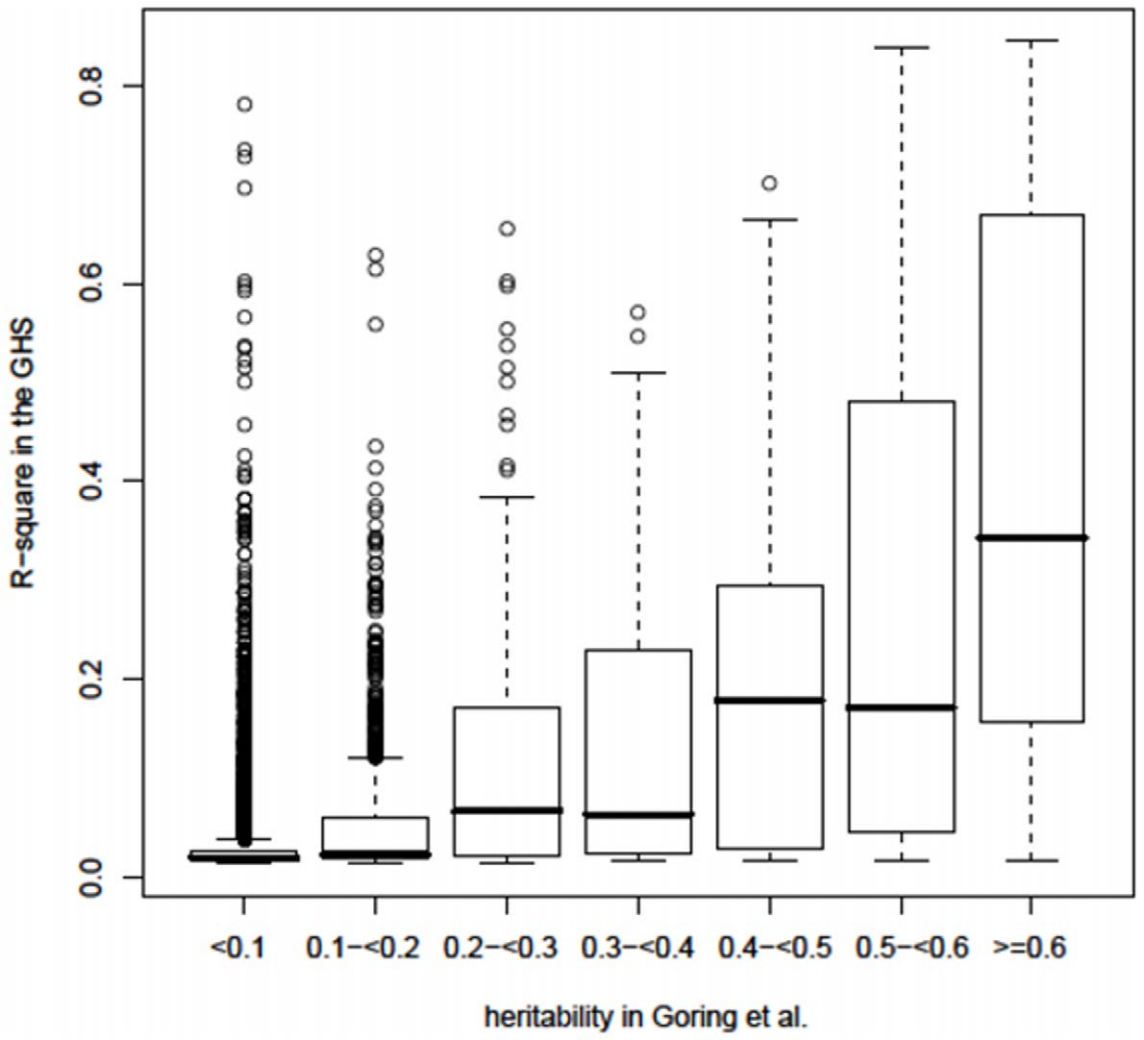
Comparison of the heritability of *cis* eQTLs estimated in the SAFHS study with the R^2^ of the corresponding *cis* eQTLs in GHS. Data were extracted from Supplementary Table 4 in Göring et al. [4] and comparisons were restricted to genes having a corresponding gene symbol in GHS. Heritability in the SAFHS was estimated by linkage analysis and accounts for the whole variability at a locus while R^2^ refers to a single eSNP (the best eSNP) and therefore underestimates the global variability affecting gene expression at a locus. The data used to generate this figure are provided in File S5. The median R^2^ was globally lower than the heritability, consistent with the fact that the R^2^ is referring to a single SNP whereas heritability reflects the whole genetic variation at a locus.

We also examined the overlap between *cis* eQTLs in GHS and *cis*-heritable eQTLs found by expression profiling of lymphocyte RNA in the San Antonio Family Heart Study (SAFHS) ^4^. Among the eQTLs with a *cis* heritability ≥0.1 in SAFHS, 62% were significantly *cis* modulated in GHS, and this proportion reached 89% for heritabilities ≥0.6 (Figure 3 , File S5).

*Trans* associations showed much weaker consistency across studies. Among the 50 eQTLs having a *trans* lod score >4.0 in SAFHS [4] with corresponding expression detected in GHS, only one, *MAPK8IP1*, was replicated in GHS (*P*<10^−300^). Replication of the *trans* associations in studies of similar power as the present one would be of interest.

### Identifying eQTLs that may result from the presence of SNPs in probe sequences

For all probes present on the *Illumina* HT12 array, a systematic search for sequence polymorphisms was undertaken, using the HapMap database as reference (Release 27; Phase II+III, Feb09, on NCBI B36 assembly and dbSNP b126). Among the 2,477 genes whose expression was associated with *cis* eSNPs, 173 (7%) were probed by one or several polymorphic sequences (180 probes) (Table S1). For 32 of these probes, the HapMap SNP was present on the Affymetrix array used in this study and for 41 other probes, the HapMap SNP had one or several perfect proxies on the array. For those eQTLs, we cannot exclude the possibility of an artefactual association due to a differential binding of the probe to its target sequence.

### Gene expression, a link between DNA sequence variability and clinical phenotypes?

A link between genetic variability and clinical phenotypes is supported in human studies by several observations relating variants in regulatory gene regions to protein phenotypes or diseases [19], [20]. However, this message-passing paradigm has never been evaluated on a genome-wide basis. We therefore tested whether monocytes gene expression might mediate the effects of loci recently identified by GWAS of cardiovascular risk factors. For each locus identified in GWAS of lipid variables [21], blood pressure (BP) [22] and body mass index (BMI) [23], we selected the lead SNP or a tag SNP having an r^2^≥0.8 with the lead SNP in GHS data. Associations between lead/tag SNPs and corresponding risk factors were checked in all GHS subjects for whom genome-wide data was available (*n* = 3,175). Most previous GWAS loci for circulating lipids were replicated in our data (Table 4) but only few of the findings of GWAS of BMI and BP were replicated (Table S2). This low replication is probably due to a lack of power, as the maximum R^2^ observed in the GWAS of BP [22] was 0.09% and the power of GHS to replicate such an association was only 38%.

**Table 4:** Loci identified in GWAS of circulating lipids – associations of lead/tag SNPs with phenotypes and expression, and of expression with phenotype in GHS.

Lead SNP in GWAS	Phenotype	Chr	Position (Mb)	Genes in region	Tag SNP in Affy 6.0 with r^2^>0.8	r^2^ between lead SNP and tag SNP	Association between tag SNP and phenotype (*P*-value)	eQTL associated with tag SNP	Association between tag SNP and eQTL (*P*-value)	Association between eQTL and phenotype (*P*-value)
rs10889353	TG	1	62.83	*DOCK7*	rs10889353	1.00	1.86E-04	*DOCK7*	7.09E-52	0.3970
**rs646776** *	**LDL**	**1**	**109.53**	***CELSR2/PSRC1/SORT1***	**rs629301**	**1.00**	**2.62E-04**	***PSRC1***	**2.34E-56**	**0.0190**
								*CELSR2*	7.56E-06	0.6195
rs693	LDL	2	21.14	*APOB*	rs693	1.00	1.61E-04	none		
rs6754295	HDL	2	21.12	*APOB*	rs673548	0.86	0.0435	none		
rs673548	TG	2	21.15	*APOB*	rs673548	1.00	4.10E-05	none		
rs780094	TG	2	27.65	*GCKR*	rs780094	1.00	3.15E-08	none		
rs6756629	LDL	2	43.98	ABCG5	rs4953023	1.00	7.87E-05	none		
rs3846662	LDL	5	74.69	*HMGCR*	rs12654264	0.84	9.26E-06	none		
rs12670798	LDL	7	21.38	*DNAH11*	none					
rs2240466	TG	7	72.3	*MLXIPL*	rs2074755	1.00	0.0015	none		
**rs2083637**	**HDL**	**8**	**19.91**	***LPL***	**rs17489282**	**1.00**	**5.91E-05**	***LPL***	**2.18E-06**	**0.0006**
rs2083637	TG	8	19.91	*LPL*	rs17489282	1.00	3.31E-07	*LPL*	2.18E-06	0.3520
rs3905000	HDL	9	104.74	*ABCA1*	rs3890182	1.00	0.33	none		
rs7395662	HDL	11	48.48	*MADD-FOLH1*	rs7395662	1.00	0.17	*MYBPC3*	1.17E-09	0.1435
								*SPI1*	4.42E-06	0.0222
rs174570	LDL	11	61.35	*FADS2/3*	rs174570	1.00	0.0386	none		
rs12272004	TG	11	116.11	*APO(A1/A4/A5/C3)*	rs10488699	1.00	1.63E-06	none		
rs1532085	HDL	15	56.47	*LIPC*	none					
rs1532624	HDL	16	55.56	*CETP*	none					
rs2271293	HDL	16	66.46	*CTCF-PRMT8*	rs2271293	1.00	0.0145	*DPEP3*	5.09E-17	0.8275
								*DUS2L*	5.15E-42	0.1410
								*GFOD2*	1.48E-17	0.6400
								*LCAT*	6.00E-06	0.3347
								*PARD6A*	7.88E-07	0.8772
								*PRMT7*	2.03E-06	0.1690
rs4939883	HDL	18	45.42	*LIPG*	rs7240405	1.00	0.0233	none		
rs2228671	LDL	19	11.07	*LDLR*	none					
rs157580	LDL	19	50.09	*TOMM40-APOE*	none					

*GWAS loci were taken from* *Table 2* *in ref. 21.*

*This SNP was also found in GWAS of CAD. The association between tag SNP and phenotype was tested in the 3,175 GHS subjects having GWV data. Association between eQTL and tag SNP or phenotype was tested in the 1,490 GHS subjects having GWE data. In bold are shown the loci for which the SNP-phenotype association found in GWAS is compatible with mediation by gene expression. Similar analyses for BMI and BP are given in Table S2.

For each GWAS locus, we examined whether the lead/tag SNP correlated with any expression trait in GHS data and when a significant association was found, we checked whether the expression trait was significantly associated with the risk factor under consideration. This analysis revealed that very few GWAS results were compatible with an effect mediated by gene expression at the locus (Table 4 and Table S2). There were, however, two exceptions: the first one concerned the *LPL* locus, where the minor allele of rs17489282 was associated with higher HDL-cholesterol (*P* = 5.91×10^−5^) and *LPL* expression (*P* = 2.18×10^−6^), while HDL-cholesterol and *LPL* expression were positively correlated (*P* = 6×10^−4^), consistent with an effect mediated by LPL; the second one concerned the association between the 1p13.3 locus and LDL-cholesterol. This locus encompasses three potential candidate genes, *CELSR2*, *PSRC1* and *SORT1*, and it has been suggested that *CELSR2* or *SORT1* could be responsible for the reported associations of this locus with LDL [3], [24], [25]. In our data, the minor allele of rs629301 (a perfect tag of the lead SNP identified by GWAS), was associated with lower LDL-cholesterol (*P* = 2.6×10^−4^) and higher *PRSC1* expression (*P* = 2.3×10^−56^) while *PRSC1* expression and LDL-cholesterol were negatively correlated (*P* = 0.019). Results for *CELSR2* were much less consistent and *SORT1*, the third gene at the locus, was not *cis*-modulated in monocytes.

**Figure 4:**
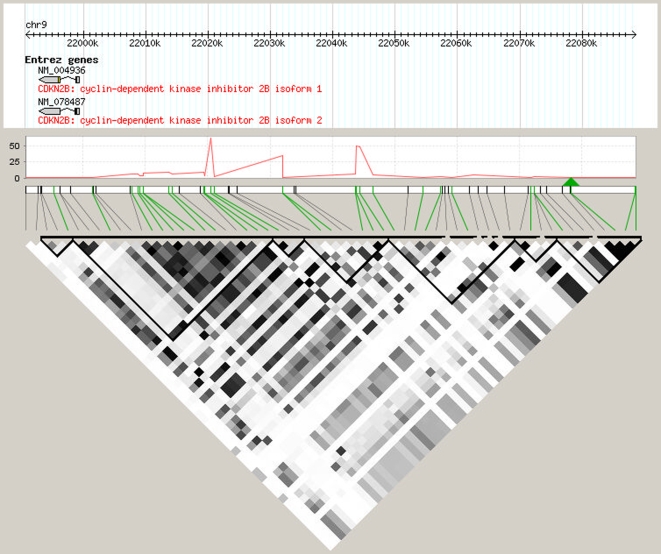
The loci affecting CDKN2B expression and CAD on chromosome 9p21 are independent. The lead SNP rs1333049 generally reported at the CAD locus was not present on the Affymetrix 6.0 array, we therefore selected its best proxy, rs10757272 (position 22078260, r^2^ = 0.9 with rs1333049), using SNAP (https://www.broadinstitute.org/mpg/snap). Positions of genotyped SNPs are shown using a green link and position of the proxy SNP, rs10757272, is represented by a green triangle. The red curve reflect the –log10(*P*-value) for the association between SNPs and *CDKN2B* expression. The LD (r^2^) between pairs of SNPs is shown at the bottom of the figure using a range of colors between white (r^2^ = 0) and black (r^2^ = 1). The *CDKN2B* and CAD-associated SNPs are located in different blocks of LD strongly suggesting that the genetic effects on *CDKN2B* expression and CAD are independent.

Several loci associated with coronary artery disease (CAD) have been identified by GWAS [26]–[29]. The strongest association involves SNPs in the 9p21 region. Recently it was reported that deletion in mice of the region orthologous to the 9p21 CAD interval in human affects the expression of the nearby *cdkn2a* and *cdkn2b* genes as well as the properties of proliferation of vascular cells [30]. The Cyclin-dependent kinase inhibitor coding genes, *CDNK2A* and *CDNK2B*, are also located close to the CAD locus in humans. *CDKN2A* expression in monocytes was not detected in our study, we therefore focused our analysis on *CDKN2B*. All SNPs available in GHS in the region encompassing the CAD locus were tested for association with the expression of *CDKN2B*. Figure 4 shows that *CDKN2B* expression was strongly associated with several SNPs located in a region upstream of the gene sequence (*P*<10^−60^). However, these SNPs were not associated with CAD (this result was obtained in a yet unpublished GWAS comparing GHS individuals to a cohort of CAD patients), whereas proxies of the CAD-associated SNPs were unrelated with *CDKN2B* expression (see legend of Figure 4 for more details). The SNPs associated with *CDKN2B* expression are located within the sequence of the non-coding alternatively spliced gene *ANRIL* (also named *CDKN2BAS*) whose implication in the association with CAD has been hypothesized [31]. Although our results are limited by the fact that neither *CDKN2A* nor *ANRIL* expressions could be evaluated, they reveal that in humans, SNPs that affect *CDKN2B* expression are different from those that are known to affect CAD risk (Figure 4).

### Expression traits associated with risk factors

To investigate gene expression in relation to risk factors (age, gender, BMI, HDL and LDL cholesterol, triglycerides, Systolic and Diastolic Blood pressure, smoking and plasma CRP), the study-wise significance threshold was set at 3.9×10^−7^ to correct for the number of risk factors (*n* = 10) and expressions (*n* = 12,808) tested. Overall, 1,662 expression traits (13.0%) were associated with at least one risk factor (Table 5 and File S6). Gender and age were the two major factors influencing expression levels (807 gene expressions were affected by gender and 396 by age). BMI, smoking and C-reactive protein (CRP) levels were also correlated with numerous expression traits (230, 294 and 328, respectively). Conversely, few associations with BP and lipids were observed (Table 5).

**Table 5:** Number of expression traits associated with risk factors and *cis* eSNPs.

Risk factor	Number of expression traits associated with the specified risk factor	Number (%) of expression traits also associated with *cis* eSNPs	Odds ratio (95% CI)*
Gender	807	230 (28.5%)	1.73 (1.47–2.03)
Age	396	94 (23.7%)	1.31 (1.03–1.66)
BMI	230	72 (31.3%)	1.92 (1.45–2.56)
HDL	9	1 (11.1%)	ND
LDL	1	0	ND
Triglycerides	9	3 (33.3%)	ND
SBP	48	6 (12.5%)	0.59 (0.25–1.40)
DBP	18	2 (11.1%)	ND
Smoking	294	126 (42.9%)	3.24 (2.56–4.10)
CRP	328	116 (35.4%)	2.34 (1.86–2.95)
All (irrespective of any association with risk factor)	12,808	2,477 (19.3%)	

Study-wise levels of significance were considered for associations of expression traits with risk factors and SNPs (3.9×10^−7^ and 5.78×10^−12^, respectively). Associations of expression traits with BMI, CRP and smoking were adjusted for age and sex, and association with HDL, LDL, triglycerides, SBP and DBP were additionally adjusted for BMI.

*Odds ratio (OR) of being influenced by a *cis* eSNP for an expression trait associated with a given risk factor. For example, gender-related expression traits have an OR of 1.73 of being influenced by *cis* eSNPs by comparison to expression traits unrelated to gender. ND: not determined because of small numbers.

### Genetic and non-genetic factors act additively on gene expression

**Figure 5:**
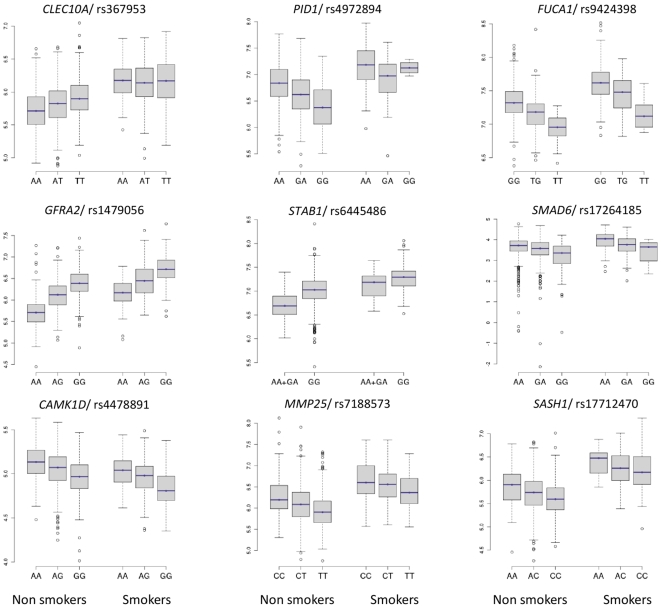
Effect of the best *cis* eSNP and smoking on expression of smoking-related eQTLs. The proportion of variability of expression explained by the best *cis* eSNP varied from 3.1% for *CLEC10A* to 27.2% for *GFRA2* while the proportion explained by smoking varied from 2.8% for *SMAD6* to 21.6% for *SASH1*. The lowest *P*-value for interaction between SNP and smoking was 0.02 for *STAB1*.

*Cis* eQTLs were over-represented among expression traits that were also affected by gender, age, BMI, smoking and CRP, with odds ratios as high as 3.24 for cigarette smoking (Table 5). This suggests that some genes are more responsive than others to the influence of multiple factors. For expression traits that were simultaneously associated with *cis* eSNPs and risk factors (*n* = 465), we determined the joint effects of the two sources of variability on expression level. For this purpose, each eQTL was modelled as a function of the best *cis*-acting eSNP, the associated risk factor and the interaction between the two. When several risk factors were associated with the same eQTL, each of them was tested separately for interaction with the corresponding SNP. The best FDR-corrected *P*-values for interaction (File S7) were 0.014 (*ISCU* expression, gender and rs4830487) and the second one was 0.042 (*HIST1H2AE* expression, smoking and rs16891378). This first genome-wide exploration of interaction between *cis* eSNPs and risk factors on gene expression therefore suggests that the two sources of variability mostly act additively on expression. This is illustrated for eQTLs affected by smoking in Figure 5. It must be noted however that despite the large size of this study, its power may nevertheless be insufficient to assess weak interaction.

### Expressions influenced by genetic and risk factors are enriched in immunity and defense genes

An ontology analysis using the Panther system demonstrated that, by reference to the list of 12,808 genes expressed in monocytes, the set of 465 expression traits affected by multiple sources of variability was enriched in “Immunity and defense” genes (69 observed/35.8 expected, *P* = 4.5×10^−6^), especially in the sub-categories of “Macrophage-mediated immunity” (18/3.6, *P* = 7.5×10^−6^).

### The variability of each risk factor can be characterized by a limited set of independent gene expressions

The preceding analyses revealed that each risk factor was associated with a large number of expression traits, thus emphasizing the multiple inter-relations existing between the transcriptomic and risk factor profiles of an individual. While it is important from a biological and mechanistic perspective to characterize at best all the genes that are influenced by a given condition, from a clinical perspective, it might be more relevant to identify a limited set of gene expressions that could efficiently discriminate individuals with different risk profiles. Indeed, because of the tight co-regulation of genes within biological systems, numerous gene expressions are inter-correlated and consequently, their association with risk factors are not independent. To account for this inter-dependency, we conducted a multivariate analysis to identify expression traits that were independently associated with each risk factor.

To obtain reliable results, we randomly divided the study population into two sub-samples of equal size which were used for screening and validation purposes respectively (see Materials and Methods). In these analyses, each risk factor was considered separately whereas all expression traits were envisaged jointly for their potential association with the risk factor, considered here as the dependent variable. The screening/validation procedure was repeated 250 times and for each risk factor, we report expression traits associated (*P*<0.01) with the risk factor in more than 25% of the replicates. This stringent approach led to the identification of 106 independent expression correlates for the ten risk factors (Table 6), a much reduced number compared to the 1,662 expression traits previously identified by the one-to-one association analysis presented in Table 5.

**Table 6:** Subsets of expression traits showing robust independent association with the different risk factors in the validation sample.

Risk factor	Median (range) of the global *P*-values across replicates*	List of gene expressions associated with risk factor after adjustment on covariates$
Gender#	All *P*<10^−100^	CCDC106, MMEL1, ANKRD57, CXCR7, FCGR2B, SOX15, FCGBP, LPXN, CD24, HOXA9, PTK6, DDX43, RAB11FIP1, PTK2, CLEC4G, ADARB1, PROK2, MYBPH, PER3, TPPP3, MPO, FAM24B, EMR3, ENOSF1, TPM2, PTTG1IP, CELSR3, CD1A, FOLR2, BOLA3, OPLAH
Age	1.1×10^−50^(2.9×10^−70^–3.6×10^−37^)	PARP3, PDGFRB, NEFH, P2RY2, SPINK2, GPER, NFKBIZ, ZSCAN18, IGLL1, BLK, ITM2C, C1RL
BMI	1.5×10^−37^(2.6×10^−50^–2.7×10^−26^)	CX3CR1, MAP3K6, FCGBP, CD209, LYPD2, VSIG4, RPGRIP1, PACAP, LGALS3BP, ELA2, CD36, ABCA1
HDL-chol	5.5×10^−10^(2.2×10^−15^–2.7×10^−3^)	PRDM1, SCD, DPEP2,TMEM43
LDL-chol	2.3×10^−3^(3.8×10^−7^–0.5)	BYSL, ABCA1
Triglycerides	4.2×10^−6^(2.1×10^−11^–0.08)	MYLIP, PHGDH, ABCA1, ELA2, ABCG1, SASH1
SBP	5.0×10^−20^(4.6×10^−27^–2.6×10^−12^)	CRIP1, GFOD1, DHRS9, NR4A2, TSC22D3, ARID5B, PAPSS2, HVCN1
DBP	1.8×10^−10^(3.6×10^−16^–1.2×10^−4^)	GFOD1, CRIP1, TPPP3, NR4A2,EMP1
CRP	6.1×10^−51^(7.3×10^−67^–2.0×10^−30^)	FAM20A, CETP, FCGBP, COL9A2, C1RL, ADM, CREB5, APBB1IP, CX3CR1, C1QB, MS4A4A, FCER1A, ALDH1A1, FLVCR2
Smoking	9.7×10^−108^(1.9×10^−128^–3.7×10^−84^)	SASH1, P2RY6, PTGDS, PID1, CYP4F22, MMP25, WWC3, FUCA1, PDE4B, STAB1, GFRA2, CLEC10A, CAMK1D, DHRS9, CNTNAP2, IQCK, ITGB7, SMAD6,

*The global *P*-value is the *P*-value obtained by comparing the model with all significant expression traits and covariates to the model with covariates only.

Expressions that are underlined are associated negatively to the risk factor (or to male gender), others are associated positively (or to female gender).

$ Covariates: age and gender for BMI, CRP and smoking; age, gender and BMI for lipids and BP.

# Gender-associated traits were selected from autosomal genes only.

#### Gender and age

Even after exclusion of sex-linked genes, gender was independently associated with the largest number of expression traits (*n* = 31) which, considered all together, contributed to a highly significant discrimination between males and females (*P*<10^−100^). By contrast the number of expression traits independently associated with age was more limited (*n* = 12).

#### BMI and CRP

Both factors were independently associated with 12 and 14 expression traits respectively. Inspection of the genes listed in Table 6 shows that several of them, including *CX3CR1*, *CD209*, *CLEC10A*, *FCER1A*, *FCGBP*, *C1RL*, *C1QB*, *CD36*, *ADM* and *VSIG4*, encode proteins involved in the differentiation or maturation of immunity-related cells and in host defence [32]–[38]. We may speculate that the variability of expression of these genes is the consequence of an already present heterogeneity of monocytes [39], [40] or reflects a particular transcription pattern that prefigures future functional changes. The example of *CX3CR1* which was positively associated with both BMI and CRP is particularly interesting as this gene encodes the fractalkine receptor whose role is essential in the migration of monocytes to sites of inflammation and injury, especially in atherosclerotic lesions [41].

#### Lipids

*ABCA1* and *ABCG1* gene expressions were both associated with circulating lipids. The proteins encoded by these genes are key players in reverse cholesterol transport and the regulation of lipid-trafficking mechanisms in macrophages respectively [42], [43]. MYLIP (Idol) is a ligase involved in the ubiquitination and degradation of LDL receptors [44], [45] and SCD is a stearoyl-CoA desaturase involved in the conversion of saturated into monounsaturated fatty acids that regulates lipid metabolism and may be modulated by dietary intake [46].

#### Blood pressure

One of the main independent correlates of SBP was *ARID5B* (*MRF2*), whose relevance in the physiology of BP regulation is supported by its role as a regulator of smooth muscle differentiation and proliferation [47]. *GFOD1*, *a*nother expression correlate of SBP and DBP is a gene of unknown function which has been associated with attention deficit hyperactivity disorder [48].

### Cigarette smoking has a major impact on gene expression and atherosclerosis

Smoking was independently associated with 18 expression traits (Table 6) which, considered all together, contributed to a highly significant discrimination between smokers and non smokers (*P*<10^−107^, R^2^>50%). Nine of these genes were modulated by *cis* eSNPs (Table 7) and as already mentioned above, the genetic and smoking effects on these gene expressions were additive (Figure 5).

**Table 7:** Smoking-related expression traits: association of expression with *cis*-acting eSNP, smoking and extent of atherosclerosis.

Smoking-related gene expressions	Association of the best *cis* eSNP with expression		Association of the best *cis* eSNP with the extent of atherosclerosis		Association of expression with smoking		Association of expression with the extent of atherosclerosis	
	SNP number	*P*-value	t- value	*P*-value	t- value	*P*-value	t- value	*P*-value
*CAMK1D*	**rs4478891**	**1.1×10^−26^**	0.7	0.51	−7.4	2.3×10^−13^	−3.1	0.002
*CLEC10A*	**rs367953**	**3.6×10^−12^**	0.5	0.65	13.0	5.4×10^−37^	−0.4	0.68
*CNTNAP2*	rs1110144	8.6×10^−12^	−1.1	0.28	6.5	7.3×10^−11^	1.7	0.082
*CYP4F22*	rs11253478	1.5×10^−6^	0.0	1.00	−17.5	1.8×10^−62^	−2.2	0.027
*DHRS9*	rs1386426	4.3×10^−7^	2.5	0.012	−6.3	3.5×10^−10^	0.2	0.82
*FUCA1*	**rs9424398**	**6.8×10^−42^**	1.0	0.34	13.7	1.4×10^−40^	2.1	0.035
*GFRA2*	**rs1479056**	**1.6×10^−108^**	0.5	0.62	13.3	3.1×10^−38^	0.8	0.44
*IQCK*	rs1879894	1.1×10^−7^	0.6	0.52	−7.6	2.7×10^−14^	−1.7	0.089
*ITGB7*	rs17080239	1.0×10^−6^	0.4	0.68	6.3	3.9×10^−10^	0.3	0.73
*MMP25*	**rs7188573**	**4.7×10^−18^**	0.0	1.00	15.6	5.4×10^−51^	**3.6**	**3.51×10^−4^**
*P2RY6*	rs3781305	9.0×10^−7^	−1.0	0.34	16.0	1.5×10^−53^	0.5	0.58
*PDE4B*	rs4352802	4.5×10^−7^	−1.2	0.24	−7.7	1.2×10^−14^	−1.8	0.077
*PID1*	**rs4972894**	**1.1×10^−25^**	−0.1	0.95	10.5	6.2×10^−25^	1.4	0.16
*PTGDS*	rs10870158	6.0×10^−9^	0.7	0.49	−14.0	3.5×10^−42^	**−5.2**	**1.77×10^−7^**
*SASH1*	**rs17712470**	**5.6×10^−14^**	0.5	0.63	20.5	5.1×10^−83^	**4.4**	**1.38×10^−5^**
*SMAD6*	**rs17264185**	**2.7×10^−15^**	−1.5	0.13	6.8	9.6×10^−12^	1.6	0.11
*STAB1*	**rs9867823**	**2.8×10^−28^**	0.4	0.68	12.3	2.9×10^−33^	0.7	0.50
*WWC3*	rs1013478	8.0×10^−6^	−1.1	0.26	10.6	1.8×10^−25^	**3.4**	**6.32×10^−4^**

Atherosclerosis was assessed by the number of carotid plaques.

Associations of expressions with smoking and number of carotid plaques were adjusted on gender and age. *Cis* eSNPs significant at a study-wise level (*P*<5.78×10^−12^) and associations with carotid plaques significant after correction for multiple testing (*n* = 18 tests) are shown in bold characters.

Among the 18 expression traits associated with smoking, *SASH1*, *P2RY6* and *PTGDS* were systematically retrieved in all screening/validation replicates (Table S3). *SASH1* is a tumor suppressor gene [49], *P2RY6* encodes a G-protein-coupled receptor involved in the proinflammatory response to UDP in monocytes [50] and *PTGDS* encodes a prostaglandin D synthase involved in smooth muscle contraction/relaxation and inhibition of platelet aggregation, two functions known to be modified by tobacco consumption. Recently, in a GWE study of leukocytes RNA, *PTGDS* and *SASH1* expressions were found associated with cotinine, a metabolite of nicotine used as a marker of tobacco exposure [51].

Cigarette smoking is a major risk factor for atherosclerosis [52]. In GHS participants, the prevalence of atherosclerotic plaques in the right and left carotid arteries, assessed by echography, was strongly increased in smokers (*P* = 9.1×10^−7^ after adjustment for age and gender). Among the 18 gene expressions independently associated with smoking, four were individually correlated with the number of carotid plaques, *PTGDS* (*P* = 1.8×10^−7^) negatively and *MMP25* (*P* = 3.5×10^−4^), *SASH1* (*P* = 1.4×10^−5^) and *WWC3* (*P* = 6.3×10^−4^) positively (Table 7). In a multivariate model including the four expression traits and smoking, as well as age and gender, *PTGDS* (*P* = 5.7×10^−4^) and *SASH1 (P = 0.012)* remained significantly associated with the number of plaques whereas *MMP25* (*P* = 0.09), *WWC3* (*P* = 0.10) and smoking (*P* = 0.5) were no longer significant, suggesting that the association between smoking and atherosclerosis was mostly reflected (or mediated) by its effect on the expression of these four genes. The fact that *PTGDS* and *SASH1* expression remained associated with carotid plaques after adjustment on smoking status may indicate a broader implication of these genes in atherosclerosis than the sole effect induced by smoking. However it is also possible that the expression of these two genes more faithfully reflects tobacco consumption than the dichotomous variable used to define smoking. This illustrates the dual aspect of the transcriptome which may be viewed either as an element in a causal chain or as reflecting ongoing processes with no implied causation. Because genetics may help to dissect causal pathways, we examined whether the best *cis* eSNPs associated with expression of the smoking-related genes were also associated with carotid atherosclerosis, but no such association was detected (Table 7).

## Discussion

This large-scale investigation of the transcriptome of monocytes in healthy individuals provides new biological insights into the mechanisms by which gene expression might contribute to disease pathogenesis. In the line of previous studies [1]–[5], we could build a detailed map of *cis*-regulated eQTLs in monocytes. Even if cell-specific eQTLs exist [53], a large fraction of them are likely to be common to other cell types, and the eQTL map provided here constitutes the most extensive one so far.

Despite the large number of eQTLs identified, the transcriptome of circulating monocytes, contrary to initial expectations [7]–[10], appeared of modest help to dissect the relationship between genome variability and complex human traits such as cardiovascular risk factors. One explanation for this finding might be that monocytes are not the most relevant cells for unravelling links between genome variation and the risk factors investigated. With regard to circulating lipids for example, only 28 of the 45 genes located in regions harbouring SNPs associated with circulating lipids in GWAS [24] were expressed in monocytes. The difficulty to corroborate the messenger paradigm in human clinical studies may also relate to the fact that the linear model of causality generally assumed to reflect the relation between genome variability, expression and phenotype may be too simplistic to account for a much more complex biological reality. The effects of genetic variants may be too weak to allow detection even in a study of this size. It is also important to keep in mind that most reported eSNPs are acting in *cis*, whereas *trans* eSNPs may actually be those that mainly drive the changes in gene expression that affect disease risk.

Most importantly, the present study highlighted for the first time the strong link existing between the transcriptome of an individual and his (her) clinical and epidemiological profile. The fact that the transcriptome tightly mirrors the variability of risk factors at a cellular level may have profound implications from a biological and clinical perspective. Until now, the traditional way of viewing the role of genes in the susceptibility to human diseases was through the effect of their variability of sequence. The present findings suggest that another important, if not greater, impact of genes on human phenotypes relates to the variability of their expression, whatever the origin of this variability. The global association observed between most cardiovascular risk factors and the transcriptome and the fact that each risk factor could be characterized by a limited and specific set of independent gene expressions further suggests that this relationship might be clinically relevant. This was particularly well illustrated by the response of the transcriptome to cigarette smoking. We showed that less than 20 genes among the 12,000 expressed in monocytes could highly discriminate smokers and non-smokers, and among them, four genes were sufficient to account for the strong association existing between smoking and atherosclerosis. Whether these genes are causally involved in the mechanisms linking smoking to the development of atherosclerotic plaques or whether they are only markers of ongoing pathological processes remains to be elucidated.

In conclusion, the variability of the transcriptome of monocytes can be viewed from two perspectives. On one hand it reflects the accumulation of effects originating from the genome and the environment and may inform on a number of ongoing processes relevant to disease. On the other hand, it may reflect or anticipate differences in monocytes biology that could have pathophysiological implications. This dual perspective suggests that a better understanding of the sources of variability of the transcriptome of monocytes and other easily accessible cells, will contribute in an important way to our understanding of complex diseases.

## Materials and Methods

### Ethic statement

The study protocol and drawing of the blood sample have been approved by the local ethics committee and by the local and federal data safety commissioners (Ethik-Kommission der Landesärztekammer Rheinland-Pfalz). All subjects included signed an informed consent.

### Study population

The Gutenberg Heart Study (GHS) is designed as a community-based, prospective, observational single-center cohort study in the Rhein-Main region in western mid-Germany. The primary aim of the study is to improve the individual cardiovascular risk prediction by identifying genetic and non genetic risk factors contributing to cardiovascular diseases, with a strong emphasis on atherosclerosis.

A sample of eligible participants was randomly drawn from the registers of the local registry offices in the city of Mainz and the district of Mainz-Bingen. This sample was stratified in a ratio of 1∶1 for gender and residence, and in equal numbers for decades of age. Inclusion criteria were an age between 35 and 74 years and a written consent; exclusion criteria were insufficient knowledge of the German language to understand explanations and instructions, and physical or psychic inability to participate in the examinations in the study center. Individuals were invited for a 5-hour baseline-examination to the study center where clinical examinations and collection of blood samples were performed. The present analysis was based on an initial sample of 3,336 subjects successively enrolled into the GHS from April 2007 to April 2008. Genomic DNA was isolated from all participants. Monocyte RNA was isolated from half of the participants recruited each day to ensure rapid sample processing and isolation of total RNA. For approximately 1,500 study participants, both DNA and RNA were available.

### Measurement and definition of cardiovascular risk factors

Blood pressure measurements were performed by an automated sphygmomanometer blood pressure meter (Omron 705CP-II, OMRON Medizintechnik Handesgesellschaft GmbH, Germany) after 5, 8 and 11 minutes of rest. The mean from the 2^nd^ and 3^rd^ standardized measurement was calculated for the systolic and diastolic blood pressure. For the anthropometric measurements, calibrated, digital scales (Seca 862, Seca Germany), a measuring stick (Seca 220, Seca, Germany) and a waist measuring tape were used. The blood sampling was carried out under fasting conditions. HDL-cholesterol, LDL-cholesterol, triglycerides and C-reactive protein (CRP) measurements were performed on an Architect c8000 by commercially available tests (CRP, Ultra HDL, Direct LDL and Triglycerides) from Abbott (www.abbottdiagnostics.de). All tests were measured under standardized conditions in an accredited laboratory of the institute of clinical chemistry and laboratory medicine at the University of Mainz. Smoking was defined by dichotomizing the population into non-smokers (never smokers and former smokers) and smokers (occasional smoker, i.e. <1 cigarette/day, and smoker, i.e. >1 cigarette/day).

### Ultrasound of the Carotid Arteries and evaluation of the number of atherosclerotic plaques

IMT was assessed with an ie33 ultrasound system (Philips, NL) using an 11 to 3 MHz linear array transducer. Experienced technologists blinded to participants' clinical data made all ultrasound measurements. The IMT was visualized bilaterally at the far wall of the CCA. In brief, a cursor representing the region of interest (10 mm) was positioned 1 cm in front of the beginning before the carotid bulb. Evaluation was performed using an automatic computerized system (Philips, NL Qlab software) and triggering was performed according to the Q wave of the ECG to enable measurement in complete relaxation of the ventricle. IMT was recorded 1 cm before the carotid bulb in a part without plaque on the left and right side. As mean IMT, the CCA was reported with the sum of IMT of the left and right side and afterwards divided by two. Plaques were defined as thickening of the IMT of at least 1.5 mm and presence was checked in all measured arteries. The number of plaques from both sides was recorded and subjects being classified as plaque positive when at least one plaque was measured on either side or plaque negative, when no plaque was recorded.

### Genotyping

For each participant genomic DNA was extracted from buffy-coats prepared from EDTA blood samples (9 mL) using the method of Miller [54]. Genotyping was performed using the *Affymetrix* Genome-Wide Human SNP Array 6.0 (http://www.affymetrix.com), as described by the *Affymetrix* user manual. Genotypes were called using the *Affymetrix* Birdseed-V2 calling algorithm and quality control was performed using GenABEL (http://mga.bionet.nsc.ru/nlru/GenABEL/). Individuals with a call rate below 97% or a too high autosomal heterozygosity (False Discovery Rate <1%) were excluded. After applying standard quality criteria (minor allele frequency >1%, genotype call rate >98% and *P*-value of deviation from Hardy-Weinberg equilibrium >10^−4^), 675,350 out of 900,392 SNPs remained for analysis.

### Separation of monocytes

Separation of monocytes was conducted within 60 min after blood collection and RNA was extracted the same day. Total RNA was isolated from monocytes using Trizol extraction and purification by silica-based columns. To separate monocytes, 8 mL blood was collected using the Vacutainer CPT Cell Preparation Tube System (BD, Heidelberg, Germany) and 400 µL Rosette Sep Monocyte Enrichment Cocktail (StemCell Technologies, Vancouver, Canada) was added immediately after blood collection. Monocytes, not labeled by antibodies, are collected as a highly enriched fraction at the interface between plasma and the density medium in the tube. After separation, cells were washed twice in ice cold PBS buffer containing 2 mM EDTA. Success of monocyte separation was controlled using an ADVIA 2120 Analyser (Siemens Healthcare Diagnostics, Eschborn, Germany) for part of the samples.

### Preparation of RNA

After separation, cells were resuspended in 1.5 mL Trizol Reagent (Invitrogen, Karlsruhe, Germany) immediately and frozen at −20°C until isolation of RNA at the same day (maximal storage time 5 h). After thawing, samples were transferred into Phase Lock Gel Tubes (Eppendorf, Hamburg, Germany), 200 mL chloroform was added and phases were separated by centrifugation at 4600 rpm for 15 min. Purification of total monocytes RNA was performed using the RNeasy Mini kit (Qiagen, Hilden, Germany) according to the manufactures' Animal Cell Spin and RNA Cleanup protocols including an additional DNase digestion step. Total RNA was eluted in 20 µL RNase-free water. Yield of RNA was checked spectrophotometrically by NanoDrop N-1000 measuring the OD260 as well as the ratio OD260 and OD280. The integrity of the total RNA was assessed through analysis on an Agilent Bioanalyzer 2100 (Agilent Technologies, Boeblingen, Germany).

### Genome-Wide Expression analysis

GWE analysis was performed on monocytes RNA samples using the *Illumina* HT-12 v3 BeadChip (http://www.Illumina.com). RNA samples were processed in batches of 96 samples. Two hundred ng of total RNA was reverse transcribed, amplified and biotinylated using the Illumina TotalPrep-96 RNA Amplification Kit (Ambion/Applied Biosystems, Darmstadt, Germany). 700 ng of each biotinylated cRNA was hybridized to a single BeadChip at 58°C for 16–18 hours. BeadChips were scanned using the *Illumina* Bead Array Reader.

### Pre-processing of expression data

The summary probe-level data delivered by the *Illumina* scanner (mean and SD computed over all beads for a particular probe) was loaded in *Beadstudio*. The pre-processing done by the *Illumina* software, at the level of the scanner and by *Beadstudio* included: correction for local background effects, removal of outlier beads, computation of average bead signal and SD for each probe and gene, calculation of detection *P*-values using negative controls present on the array, quantile normalization across arrays, check of outlier samples using a clustering algorithm, check of positive controls. Analyses were carried out on the mean level for all probes in each gene. To stabilise variance across expression levels, we applied an arcsinh transformation to the expression data [55]. Compared to a log transformation, this transformation has the advantage not to discard negative expression values which can occur in *Illumina* data.

The *Illumina* HT-12 BeadChip included 37,804 genes (some probes being not assigned to RefSeq genes). A gene was declared significantly expressed in the dataset, i.e. expressed above background (as measured by the negative controls present on each array), when the detection *P*-value calculated by *Beadstudio* was <0.05 in more that 5% of the samples. This resulted in 22,305 genes considered as being significantly expressed in our dataset. After removing 8,058 putative and/or non well characterized genes, i.e. gene names starting by KIAA (*n* = 165), FLJ (*n* = 214), HS (*n* = 4,262), C*x*orf (*n* = 842), MGC (*n* = 72), LOC (*n* = 2,503), 12,808 well characterized detected genes remained for analysis.

### Genome wide association analysis

To test all associations between SNPs and expressions in a reasonable amount of time, a C script calling the GNU Scientific Library (GSL) “TAMU ANOVA” (www.stat.tamu.edu/~aredd/tamuanova/) was written. For all significant associations, results were checked against the R-lm library [56]. When the numbers of homozygotes for the minor allele of a SNP was lower than 30, they were grouped with heterozygotes. We used a family-wise error rate of 0.05 corrected for the number of tested SNP x expression associations, which corresponds to declare significant any association with a *P*-value <5.78×10^−12^. To increase robustness, associations significant by ANOVA were further checked by a Kruskall-Wallis (KW) test and only associations with a *P*-value <10^−10^ by the KW test were retained. *P*-values given in the results and in the GHS-Express database are those obtained by ANOVA. For SNPs on chromosome X, associations with gene expression were assessed separately in women and men and the *P*-values were combined using the Fisher method [57].

### Association of gene expressions with CVD risk factors

The relationship between gene expression and each CHD risk factor was tested by a linear regression model using R-lm, with gene expression as the dependent variable. Association with age was adjusted for gender while association with other risk factors were adjusted for gender, age and, if specified, BMI. A square root transformation was applied to CRP levels to remove positive skewness. A study-wise statistical significance threshold of 3.9×10^−7^ was used to correct for the number of tests (10 risk factors ×12,808 gene expressions). For each expression trait that was associated with a risk factor and also affected by cis eSNPs, we tested the interaction between the risk factor and the best cis eSNP on expression in a regression model.

### Global assessment of associations between the monocyte transcriptome and CVD risk factors

The goal of this analysis was to identify subsets of expression traits independently associated with each risk factor. To increase the robustness of the analysis the population was randomly divided into 2 sub-samples of equal size which were used for screening and validation purpose respectively. The screening step was focused on the subsets of expression traits that were associated with each covariate-adjusted risk factor in univariate analysis at *P*<3.9×10^−6^ (Bonferroni correction for 12,808 expressions). Each risk factor and corresponding subset of expression traits were included as dependent and predictor variables respectively in a forward stepwise regression model to identify expression traits that were independently associated with the risk factor (*P*<0.01). Gene expressions selected at the screening step were then jointly tested in the validation sample for association with the risk factor by multiple regression analysis. This screening/validation procedure was repeated 250 times and for each risk factor, expression traits associated (*P*<0.01) with the risk factor in more than 25% of the replicates are reported.

### Power of the SNP-expression association analysis

Power was calculated using the program Quanto (http://hydra.usc.edu/GxE/). Assuming a quantitative expression trait with mean 0 and SD 1, a sample size of 1,490 subjects, a type I error of 5.78×10^−12^ and an additive allele effect, the study had a 82% power to detect the effect of a SNP explaining 4% of gene expression.

### Functional classification of genes

An ontology analysis was performed using the Panther database (http://www.pantherdb.org/). Lists or sublists of genes involved in associations with eSNPs or risk factors were compared to the background list of the 12,808 genes. The *P*-value calculated by the binomial statistic and Bonferroni-corrected was used.

### Quality checking and exclusion of outliers

Population stratification and quality of genotypes and expression data were tested extensively and outliers were excluded on the basis of multidimensional scaling analysis (see Methods S2)

### GHS_Express

A downloadable SQL database compiling the results of the various associations tested is available online (http://genecanvas.ecgene.net/uploads/ForReview/), see also Methods S1. This database can be used to test specific hypotheses.

## Supporting Information

Table S1 Characterization of polymorphic probes in eQTLs.
(0.33 MB DOC)

Table S2 Loci identified in GWAS of BMI and BP - associations of lead/tag SNPs with phenotypes and expressions, and of expressions with phenotype in GHS.
(0.07 MB DOC)

Table S3 Sets of gene expressions robustly and independently associated with each risk factor in the validation samples.
(0.21 MB DOC)

Methods S1 GHS-Express database.
(0.18 MB DOC)

Methods S2 Data quality checking.
(0.21 MB DOC)

File S1 GHS - Characteristics of cis eQTLs.
(0.40 MB XLS)

File S2 Comparison cis eQTL in GHS and the study of Stranger et al.
(0.10 MB XLS)

File S3 Comparison cis eQTL in GHS and the study of Dixon et al.
(0.14 MB XLS)

File S4 Comparison cis eQTL in GHS and the study of Schadt et al.
(0.28 MB XLS)

File S5 Comparison cis eQTL in GHS and the study of Goring et al.
(0.83 MB XLS)

File S6 GHS - Expression traits associated with risk factors.
(0.29 MB XLS)

File S7 GHS - Interaction cis eSNPs with risk factors.
(0.12 MB XLS)
