# Supplementary material for: Genetics and Beyond – The Transcriptome of Human Monocytes and Disease Susceptibility
Source: PLoS One. 2010 May 18;5(5):e10693. doi: 10.1371/journal.pone.0010693 (PMC2872668; doi:10.1371/journal.pone.0010693)
Supplement: Table S1 — Characterization of polymorphic probes in eQTLs. (0.33 MB DOC) [file pone.0010693.s001.doc]

### Table S1. Characterization of polymorphic probes in eQTLs

| **Gene Symbol** | **Chromo-some** | **Probe Id** | **Probe Start** | **Probe End** | **Nb. of HapMap SNPs** | **HapMap SNP** | **Position of HapMap SNP** | **Nb of Affy SNPs** | **Affy SNP** | **Position of Affy SNP** | **Proxies of HapMap SNPs present on Affy array** |
| --- | --- | --- | --- | --- | --- | --- | --- | --- | --- | --- | --- |
| ABHD2 | 15 | ILMN_2403446 | 87539746 | 87539795 | 1 | rs8029539 | 87539758 | 0 | NA | NA |  |
| ACADM | 1 | ILMN_2053679 | 76001667 | 76001716 | 1 | rs8763 | 76001708 | 0 | NA | NA |  |
| ACP2 | 11 | ILMN_2104830 | 47217622 | 47217671 | 1 | rs4640 | 47217647 | 1 | rs4640 | 47217647 |  |
| ADARB2 | 10 | ILMN_1749493 | 1218169 | 1218218 | 1 | rs1046914 | 1218206 | 0 | NA | NA |  |
| ADCY4 | 14 | ILMN_2148944 | 23857547 | 23857596 | 1 | rs3181386 | 23857555 | 0 | NA | NA |  |
| AGTRAP | 1 | ILMN_2307656 | 11733061 | 11733110 | 1 | rs8617 | 11733092 | 0 | NA | NA | rs6660106-rs6661241 |
| ALDH8A1 | 6 | ILMN_2365176 | 135280615 | 135280664 | 1 | rs9494118 | 135280624 | 0 | NA | NA |  |
| AMACR | 5 | ILMN_2367172 | 34023932 | 34023981 | 1 | rs16892066 | 34023973 | 0 | NA | NA |  |
| ANXA6 | 5 | ILMN_2326591 | 150460696 | 150460745 | 1 | rs11960458 | 150460713 | 0 | NA | NA |  |
| ARRDC4 | 15 | ILMN_2184064 | 96317610 | 96317659 | 1 | rs1043374 | 96317626 | 1 | rs1043374 | 96317626 |  |
| ARSA | 22 | ILMN_2047240 | 49410342 | 49410391 | 1 | rs6151429 | 49410343 | 1 | rs6151429 | 49410343 |  |
| ATE1 | 10 | ILMN_2381769 | 123492928 | 123492977 | 1 | rs7919858 | 123492961 | 0 | NA | NA |  |
| ATP5A1 | 18 | ILMN_2341363 | 41920138 | 41920187 | 1 | rs11541934 | 41920164 | 0 | NA | NA |  |
| ATPAF1 | 1 | ILMN_2395055 | 46871075 | 46871124 | 1 | rs13374271 | 46871082 | 0 | NA | NA |  |
| BAG5 | 14 | ILMN_2361695 | 103093426 | 103093475 | 1 | rs7693 | 103093457 | 1 | rs7693 | 103093457 |  |
| BARD1 | 2 | ILMN_2074258 | 215301702 | 215301751 | 1 | rs13389324 | 215301714 | 0 | NA | NA |  |
| BEX4 | X | ILMN_2351638 | 102358026 | 102358075 | 1 | rs3180254 | 102358043 | 0 | NA | NA |  |
| BIN1 | 2 | ILMN_2309245 | 127522387 | 127522436 | 1 | rs762740 | 127522419 | 0 | NA | NA |  |
| BRCA1 | 17 | ILMN_2311089 | 38449934 | 38449983 | 1 | rs12516 | 38449934 | 0 | NA | NA | rs8176318-rs4793197-rs8176126-rs4520874-rs4793229 |
| BRWD2 | 10 | ILMN_2086222 | 122658780 | 122658829 | 1 | rs1045179 | 122658791 | 0 | NA | NA | rs1530116-rs11199636-rs4751810-rs7094695-rs7911084-rs6585674-rs12415091-rs754869-rs4372362-rs3758510-rs3758509-rs3758507-rs11199602 |
| BTN3A2 | 6 | ILMN_1676528 | 26486092 | 26486141 | 1 | rs9104 | 26486128 | 0 | NA | NA | rs12203176-rs9461245 |
| CASP7 | 10 | ILMN_2362974 | 115479121 | 115479170 | 2 | rs2227310-rs2227309 | 115479142-115479157 | 1 | rs2227310 | 115479142 | NA/rs3814231 |
| CAST | 5 | ILMN_2322806 | 96135867 | 96135916 | 1 | rs3797810 | 96135885 | 0 | NA | NA |  |
| CAT | 11 | ILMN_2151739 | 34449993 | 34450042 | 1 | rs2266629 | 34450021 | 0 | NA | NA |  |
| CCL5 | 17 | ILMN_2098126 | 31222639 | 31222688 | 1 | rs28914814 | 31222687 | 0 | NA | NA |  |
| CD200R1 | 3 | ILMN_2408112 | 114130492 | 114130541 | 1 | rs9826308 | 114130522 | 0 | NA | NA |  |
| CD52 | 1 | ILMN_2208903 | 26519273 | 26519322 | 2 | rs1071849-rs17645 | 26519313-26519317 | 0 | NA | NA | rs3795684-rs3795684 |
| CEP192 | 18 | ILMN_1703754 | 13114633 | 13114682 | 1 | rs9961213 | 13114655 | 0 | NA | NA |  |
| CHCHD2 | 7 | ILMN_2191681 | 56136866 | 56136915 | 1 | rs8406 | 56136913 | 0 | NA | NA |  |
| CLEC12A | 12 | ILMN_2403228 | 10029119 | 10029168 | 1 | rs1349027 | 10029153 | 0 | NA | NA |  |
| CMAH | 6 | ILMN_2086612 | 25192695 | 25192744 | 1 | rs303006 | 25192697 | 0 | NA | NA |  |
| COMMD5 | 8 | ILMN_2235454 | 146049070 | 146049119 | 1 | rs2727227 | 146049076 | 0 | NA | NA |  |
| CPNE1 | 20 | ILMN_2276000 | 33704895 | 33704944 | 1 | rs17092928 | 33704944 | 0 | NA | NA |  |
| CPVL | 7 | ILMN_2400759 | 29002023-29036718 | 29002023-29036766 | 1 | NA-rs7313 | NA-29036734 | 0 | NA | NA |  |
| CTBS | 1 | ILMN_2144574 | 84801627 | 84801676 | 1 | rs15911 | 84801665 | 0 | NA | NA |  |
| CTNNA1 | 5 | ILMN_2230902 | 138298393 | 138298442 | 1 | rs17031 | 138298441 | 1 | rs17031 | 138298441 |  |
| CTSC | 11 | ILMN_2242463 | 87666842 | 87666891 | 1 | rs3888798 | 87666857 | 1 | rs3888798 | 87666857 |  |
| CTSH | 15 | ILMN_1752451 | 77001425 | 77001474 | 1 | rs3128 | 77001447 | 0 | NA | NA |  |
| DAP | 5 | ILMN_2112493 | 10732621 | 10732670 | 1 | rs5745304 | 10732634 | 0 | NA | NA |  |
| DDR1 | 6 | ILMN_2360054 | 30975476 | 30975525 | 1 | rs1049633 | 30975506 | 0 | NA | NA | rs3132581-rs3130782 |
| DEF6 | 6 | ILMN_2145280 | 35397424 | 35397473 | 1 | rs707969 | 35397424 | 1 | rs707969 | 35397424 |  |
| DGUOK | 2 | ILMN_2312897 | 74039372 | 74039421 | 1 | rs4777 | 74039384 | 1 | rs4777 | 74039384 |  |
| DNAJC15 | 13 | ILMN_1812666 | 42581232 | 42581281 | 1 | rs9590742 | 42581244 | 0 | NA | NA |  |
| DSTN | 20 | ILMN_1794294 | 17520652 | 17520701 | 1 | rs6080746 | 17520666 | 0 | NA | NA |  |
| DYNC2LI1 | 2 | ILMN_2281734 | 43886100 | 43886149 | 1 | rs1432278 | 43886126 | 0 | NA | NA |  |
| EEF1G | 11 | ILMN_2262288 | 62091456 | 62091505 | 1 | rs6591717 | 62091484 | 0 | NA | NA |  |
| EFHB | 3 | ILMN_2199409 | 19896127 | 19896176 | 1 | rs11917204 | 19896153 | 0 | NA | NA |  |
| EIF5 | 14 | ILMN_2318430 | 102880716 | 102880765 | 1 | rs9718 | 102880719 | 0 | NA | NA |  |
| EMR3 | 19 | ILMN_2348487 | 14591069 | 14591118 | 1 | rs2482 | 14591109 | 0 | NA | NA | rs11879893 |
| ENPP2 | 8 | ILMN_2373791 | 120639027 | 120639076 | 1 | rs2305129 | 120639058 | 0 | NA | NA | rs3816330 |
| EPHB2 | 1 | ILMN_2367384 | 23114144 | 23114193 | 1 | rs2295022 | 23114188 | 0 | NA | NA |  |
| ERCC8 | 5 | ILMN_2348146 | 60235284-60236376 | 60235300-60236408 | 1 | NA-rs167037 | NA-60236408 | 0 | NA | NA |  |
| ERCC8 | 5 | ILMN_1746661 | 60235079 | 60235128 | 1 | rs4647102 | 60235120 | 0 | NA | NA | rs4235483-rs158570-rs158572-rs1382914-rs290506-rs162242 |
| EXOC2 | 6 | ILMN_2065783 | 430604 | 430653 | 1 | rs6597054 | 430620 | 1 | rs6597054 | 430620 |  |
| FAIM | 3 | ILMN_2351548 | 139830660-139834462 | 139830688-139834482 | 1 | rs13043-NA | 139830678-NA | 1 | rs13043-NA | 139830678-NA |  |
| FAM110A | 20 | ILMN_2323944 | 774496 | 774545 | 1 | rs2282051 | 774513 | 0 | NA | NA |  |
| FAM36A | 1 | ILMN_1808584 | 243074707 | 243074756 | 1 | rs11579106 | 243074738 | 0 | NA | NA |  |
| FANCD2 | 3 | ILMN_2235137 | 10118398 | 10118447 | 1 | rs2600013 | 10118432 | 0 | NA | NA |  |
| FCRL6 | 1 | ILMN_2074762 | 158052005 | 158052054 | 1 | rs4443889 | 158052049 | 1 | rs4443889 | 158052049 |  |
| FEZ2 | 2 | ILMN_2403946 | 36633209 | 36633258 | 1 | rs11885432 | 36633254 | 0 | NA | NA |  |
| FOXK2 | 17 | ILMN_2310685 | 78154897 | 78154946 | 1 | rs3794713 | 78154905 | 0 | NA | NA |  |
| FVT1 | 18 | ILMN_2154052 | 59149381 | 59149430 | 1 | rs6810 | 59149381 | 0 | NA | NA |  |
| FXYD5 | 19 | ILMN_2309848 | 40352545 | 40352594 | 1 | rs1046345 | 40352592 | 0 | NA | NA |  |
| GALC | 14 | ILMN_2415572 | 87501642 | 87501691 | 1 | rs12888666 | 87501651 | 0 | NA | NA | rs11621530-rs12881373 |
| GPATCH4 | 1 | ILMN_2383305 | 154831173 | 154831222 | 2 | rs10757-rs4661047 | 154831192-154831201 | 0 | NA | NA | rs16837530-rs6668178-rs942960-rs7355216-rs11264506-rs4661194-rs7512734-rs4661200-rs4661202 |
| GPATCH4 | 1 | ILMN_2383306 | 154831238 | 154831287 | 1 | rs3795732 | 154831264 | 0 | NA | NA | rs879461 |
| GRINA | 8 | ILMN_2370872 | 145139423 | 145139472 | 1 | rs9100 | 145139455 | 0 | NA | NA | rs6985603 |
| GRK4 | 4 | ILMN_2375739 | 3008927 | 3008976 | 1 | rs1801058 | 3008948 | 1 | rs1801058 | 3008948 |  |
| GSTZ1 | 14 | ILMN_2381296 | 76867617 | 76867666 | 1 | rs3742739 | 76867628 | 0 | NA | NA |  |
| GTF2H1 | 11 | ILMN_2157957 | 18344968 | 18345017 | 1 | rs4150685 | 18345017 | 0 | NA | NA |  |
| HLA-DOA | 6 | ILMN_1659075 | 33080184 | 33080233 | 1 | rs3130602 | 33080185 | 1 | rs3130602 | 33080185 |  |
| HLA-DRA | 6 | ILMN_2157441 | 32520491 | 32520540 | 1 | rs7195 | 32520517 | 1 | rs7195 | 32520517 |  |
| HLA-F | 6 | ILMN_2186806 | 29801794-29801935 | 29801799-29801978 | 1 | NA-rs1736922 | NA-29801948 | 0 | NA | NA |  |
| HPS1 | 10 | ILMN_2411658 | 100179386-100180318 | 100179389-100180363 | 1 | NA-rs11189601 | NA-100180344 | 0 | NA | NA |  |
| IFT88 | 13 | ILMN_2373755 | 20117096-20126015 | 20117097-20126062 | 1 | NA-rs9552254 | NA-20126049 | 0 | NA | NA |  |
| IL4R | 16 | ILMN_1691881 | 27273891 | 27273940 | 1 | rs3024691 | 27273899 | 0 | NA | NA |  |
| INCENP | 11 | ILMN_1698171 | 61676714 | 61676763 | 1 | rs1675066 | 61676759 | 0 | NA | NA |  |
| IPO8 | 12 | ILMN_1753164 | 30673525 | 30673574 | 1 | rs7326 | 30673568 | 0 | NA | NA | rs7327-rs6487923-rs2289300-rs4143105-rs10771748-rs3910561-rs6487925-rs10771755-rs10743728 |
| ITM2A | X | ILMN_2076600 | 78503309-78503482 | 78503330-78503509 | 1 | NA-rs3180834 | NA-78503508 | 0 | NA | NA |  |
| KCTD12 | 13 | ILMN_2229649 | 76352471 | 76352520 | 1 | rs6835 | 76352482 | 1 | rs6835 | 76352482 |  |
| KLHL5 | 4 | ILMN_2342437 | 38793305-38799025 | 38793345-38799033 | 1 | rs3733276-NA | 38793306-NA | 0 | NA | NA | rs6829450-rs7685468-rs12499266-rs9996285/NA |
| KLRC2 | 12 | ILMN_1707328 | 10477804-10478352 | 10477808-10478396 | 3 | NA-rs4587808-rs3908178-rs1141715 | NA-10478363-10478367-10478378 | 0 | NA | NA |  |
| KLRC2 | 12 | ILMN_2059357 | 10474629 | 10474678 | 1 | rs2682508 | 10474632 | 0 | NA | NA |  |
| KRIT1 | 7 | ILMN_2323418 | 91680483 | 91680532 | 1 | rs11542682 | 91680490 | 0 | NA | NA | rs11769780 |
| LASS5 | 12 | ILMN_2208495 | 48810125 | 48810174 | 1 | rs2293556 | 48810131 | 0 | NA | NA |  |
| LEMD3 | 12 | ILMN_2183938 | 63928218 | 63928267 | 1 | rs2888 | 63928252 | 0 | NA | NA |  |
| LGALS8 | 1 | ILMN_2266214 | 234748352 | 234748401 | 1 | rs1266380 | 234748373 | 1 | rs1266380 | 234748373 |  |
| LGALS9 | 17 | ILMN_2412214 | 22998484 | 22998533 | 1 | rs361499 | 22998533 | 0 | NA | NA |  |
| LIG3 | 17 | ILMN_2373335 | 30353808 | 30353857 | 1 | rs4986974 | 30353818 | 0 | NA | NA |  |
| LRRC61 | 7 | ILMN_1669722 | 149665841 | 149665890 | 1 | rs3735168 | 149665884 | 0 | NA | NA |  |
| MAK | 6 | ILMN_2209115 | 10871282 | 10871331 | 1 | rs526495 | 10871298 | 0 | NA | NA |  |
| MAN2C1 | 15 | ILMN_2111739 | 73435608-73435695 | 73435613-73435738 | 2 | NA-rs3803466-rs3803467 | NA-73435703-73435733 | 0 | NA | NA |  |
| MANBAL | 20 | ILMN_2252554 | 35360602 | 35360651 | 1 | rs6131016 | 35360640 | 0 | NA | NA |  |
| MARCO | 2 | ILMN_1731503 | 119468445 | 119468494 | 1 | rs34536804 | 119468467 | 0 | NA | NA |  |
| MCFD2 | 2 | ILMN_2202790 | 46982769 | 46982818 | 1 | rs8861 | 46982788 | 0 | NA | NA | rs17035813 |
| MRPL52 | 14 | ILMN_2311041 | 22373917 | 22373966 | 1 | rs3751488 | 22373934 | 1 | rs3751488 | 22373934 |  |
| MSH5 | 6 | ILMN_2325394 | 31837700-31837868 | 31837711-31837905 | 1 | NA-rs1802127 | NA-31837904 | 1 | NA-rs1802127 | NA-31837904 |  |
| MTRR | 5 | ILMN_2377977 | 7945973 | 7946022 | 1 | rs16879355 | 7946013 | 0 | NA | NA |  |
| MYO10 | 5 | ILMN_2232712 | 16718478 | 16718527 | 1 | rs16868940 | 16718503 | 0 | NA | NA |  |
| MYO19 | 17 | ILMN_1750711 | 31943223 | 31943272 | 1 | rs8882 | 31943268 | 0 | NA | NA |  |
| N4BP1 | 16 | ILMN_2201966 | 47130290 | 47130339 | 1 | rs1224 | 47130318 | 0 | NA | NA | rs9924078-rs9933187-rs9922734-rs8052640-rs8059905-rs2129243-rs9937610-rs7194570-rs6500402-rs7199206-rs7190106 |
| NANP | 20 | ILMN_2064132 | 25543104 | 25543153 | 1 | rs958075 | 25543123 | 1 | rs958075 | 25543123 |  |
| NAPB | 20 | ILMN_2181125 | 23303396 | 23303445 | 1 | rs8615 | 23303423 | 1 | rs8615 | 23303423 |  |
| NBN | 8 | ILMN_2358041 | 91014768 | 91014817 | 1 | rs10464867 | 91014774 | 0 | NA | NA | rs13312971-rs10504884 |
| NEBL | 10 | ILMN_1808824 | 21110350 | 21110399 | 1 | rs11012338 | 21110399 | 0 | NA | NA | rs1467642 |
| ODF2L | 1 | ILMN_2325028 | 86592810 | 86592859 | 1 | rs1182098 | 86592855 | 0 | NA | NA | rs272493-rs272500-rs272514 |
| OGFRL1 | 6 | ILMN_2095759 | 72063202-72067810 | 72063241-72067819 | 1 | rs2273888-NA | 72063233-NA | 1 | rs2273888-NA | 72063233-NA |  |
| PCBD1 | 10 | ILMN_1795906 | 72315599 | 72315648 | 1 | rs11554325 | 72315605 | 0 | NA | NA |  |
| PCBD1 | 10 | ILMN_1813456 | 72312184 | 72312233 | 1 | rs2630331 | 72312202 | 0 | NA | NA |  |
| PIP5K2A | 10 | ILMN_2152465 | 22866077 | 22866126 | 1 | rs1053454 | 22866099 | 0 | NA | NA |  |
| PLAUR | 19 | ILMN_2374340 | 48848309 | 48848358 | 1 | rs2302524 | 48848312 | 0 | NA | NA |  |
| PPARG | 3 | ILMN_2364384 | 12450512 | 12450561 | 1 | rs3856806 | 12450557 | 0 | NA | NA |  |
| PPFIA1 | 11 | ILMN_2382126 | 69901832 | 69901881 | 1 | rs12172761 | 69901856 | 0 | NA | NA |  |
| PRKAR1A | 17 | ILMN_2389590 | 64040350 | 64040399 | 1 | rs6958 | 64040373 | 0 | NA | NA | rs2302234 |
| PRKAR1A | 17 | ILMN_2277077 | 64019822 | 64019871 | 1 | rs8080306 | 64019838 | 0 | NA | NA |  |
| PSMB9 | 6 | ILMN_2376108 | 32933044 | 32933093 | 1 | rs17587 | 32933068 | 0 | NA | NA |  |
| PTGS2 | 1 | ILMN_2054297 | 184907659 | 184907708 | 1 | rs689470 | 184907681 | 0 | NA | NA |  |
| PTPN22 | 1 | ILMN_2246328 | 114158149 | 114158198 | 1 | rs3811021 | 114158186 | 0 | NA | NA | rs3765598 |
| RAB40B | 17 | ILMN_2230566 | 78208302 | 78208351 | 1 | rs7224226 | 78208329 | 0 | NA | NA |  |
| RAD21 | 8 | ILMN_2221006 | 117927527 | 117927576 | 2 | rs14795-rs4570 | 117927531-117927570 | 0 | NA | NA | rs16889218/NA |
| REPS2 | X | ILMN_2405797 | 17063402-17066900 | 17063450-17066900 | 1 | rs12847967-NA | 17063414-NA | 0 | NA | NA |  |
| RIPK5 | 1 | ILMN_2352023 | 203378372 | 203378421 | 1 | rs2836 | 203378384 | 1 | rs2836 | 203378384 |  |
| RNF14 | 5 | ILMN_2351241 | 141348057 | 141348106 | 1 | rs433623 | 141348082 | 0 | NA | NA | rs403075-rs7408 |
| ROD1 | 9 | ILMN_2117223 | 114020788 | 114020837 | 1 | rs6575 | 114020788 | 1 | rs6575 | 114020788 |  |
| RPA1 | 17 | ILMN_2049642 | 1747890 | 1747939 | 2 | rs17734-rs1131636 | 1747894-1747939 | 0 | NA | NA |  |
| RPL14 | 3 | ILMN_2404850 | 40478782 | 40478831 | 1 | rs11539046 | 40478813 | 0 | NA | NA | rs2276871-rs11714912-rs6801859-rs11707278-rs4571217-rs1047855-rs9848083-rs4973898-rs9857824-rs9332450-rs7648952-rs17078941-rs7625736-rs7631893-rs897117-rs17078813-rs1119179-rs10510708-rs6599094 |
| RPS23 | 5 | ILMN_1772459 | 81605044 | 81605093 | 1 | rs3738 | 81605049 | 0 | NA | NA |  |
| RPS6KA1 | 1 | ILMN_2251452 | 26729039-26736003 | 26729061-26736029 | 1 | rs11800553-NA | 26729049-NA | 0 | NA | NA |  |
| RPS6KB2 | 11 | ILMN_2364357 | 66959398 | 66959447 | 2 | rs10274-rs3815364 | 66959402-66959423 | 0 | NA | NA | NA/rs2276020 |
| RUNX2 | 6 | ILMN_1716651 | 45626169 | 45626218 | 1 | rs1200428 | 45626180 | 0 | NA | NA |  |
| SCN9A | 2 | ILMN_2169692 | 166763260 | 166763309 | 1 | rs16851759 | 166763299 | 0 | NA | NA |  |
| SCP2 | 1 | ILMN_1674955 | 53232031 | 53232080 | 1 | rs6657017 | 53232049 | 0 | NA | NA | rs7930896-rs7529265-rs7549603-rs17107624-rs17107654-rs12568971 |
| SF3A1 | 22 | ILMN_1697286 | 29058343 | 29058392 | 1 | rs10376 | 29058356 | 0 | NA | NA | rs4339043 |
| SFXN1 | 5 | ILMN_2205935 | 174887440 | 174887489 | 1 | rs156373 | 174887467 | 0 | NA | NA | rs267399-rs157476 |
| SIGLEC7 | 19 | ILMN_2409384 | 56348242 | 56348291 | 1 | rs273662 | 56348266 | 0 | NA | NA | rs273663-rs183893-rs273666-rs273654-rs273655 |
| SLC35B4 | 7 | ILMN_1697959 | 133625015 | 133625064 | 1 | rs8134 | 133625059 | 0 | NA | NA | rs2241333-rs2241334-rs2241335-rs2241336-rs2598291 / rs9500867-rs7749235-rs2270172-rs11757306-rs6928738-rs9262551 |
| SLC39A8 | 4 | ILMN_2233539 | 103402198 | 103402247 | 1 | rs9331 | 103402241 | 1 | rs9331 | 103402241 |  |
| SLC41A3 | 3 | ILMN_2356111 | 127207987 | 127208036 | 1 | rs9833685 | 127208024 | 0 | NA | NA |  |
| SLC45A4 | 8 | ILMN_1745778 | 142286571 | 142286620 | 1 | rs2474 | 142286577 | 0 | NA | NA |  |
| SMAD7 | 18 | ILMN_2203891 | 44700792 | 44700841 | 1 | rs8088297 | 44700838 | 0 | NA | NA |  |
| SMC6 | 2 | ILMN_1654001 | 17708749 | 17708798 | 1 | rs2710673 | 17708777 | 1 | rs2710673 | 17708777 |  |
| SOD2 | 6 | ILMN_2406501 | 160033762 | 160033811 | 1 | rs1804451 | 160033777 | 0 | NA | NA |  |
| SP4 | 7 | ILMN_1721081 | 21520130 | 21520179 | 1 | rs2285941 | 21520146 | 0 | NA | NA |  |
| SPHK1 | 17 | ILMN_2357134 | 71895257-71895263 | 71895262-71895306 | 1 | NA-rs346801 | NA-71895273 | 0 | NA | NA |  |
| SRA1 | 5 | ILMN_2062754 | 139910056 | 139910105 | 1 | rs6889768 | 139910061 | 0 | NA | NA |  |
| ST6GALNAC4 | 9 | ILMN_2413064 | 129710209 | 129710258 | 1 | rs1127024 | 129710253 | 0 | NA | NA |  |
| STYXL1 | 7 | ILMN_2210729 | 75468167 | 75468216 | 1 | rs8565 | 75468210 | 0 | NA | NA | rs6978677 |
| TFIP11 | 22 | ILMN_2408102 | 25218084 | 25218133 | 1 | rs3177310 | 25218091 | 0 | NA | NA |  |
| THG1L | 5 | ILMN_2093720 | 157099139 | 157099188 | 1 | rs3194515 | 157099176 | 1 | rs3194515 | 157099176 |  |
| TMEM107 | 17 | ILMN_2413330 | 8017611 | 8017660 | 1 | rs11655582 | 8017636 | 0 | NA | NA |  |
| TMEM86B | 19 | ILMN_2049417 | 60429973 | 60430022 | 1 | rs3826884 | 60429993 | 1 | rs3826884 | 60429993 |  |
| TMEM9B | 11 | ILMN_2100815 | 8925505 | 8925554 | 1 | rs11605811 | 8925529 | 0 | NA | NA |  |
| TMPRSS3 | 21 | ILMN_2358474 | 42676228 | 42676277 | 1 | rs2839500 | 42676236 | 0 | NA | NA |  |
| TOMM7 | 7 | ILMN_2087060 | 22819163 | 22819212 | 1 | rs1054471 | 22819168 | 0 | NA | NA |  |
| TPP2 | 13 | ILMN_2128358 | 102129197 | 102129246 | 1 | rs1062536 | 102129212 | 0 | NA | NA |  |
| TRAF1 | 9 | ILMN_1698218 | 122705105 | 122705154 | 2 | rs7037154-rs10985085 | 122705112-122705118 | 0 | NA | NA | NA/rs7867876-rs10985112 |
| TRAPPC6B | 14 | ILMN_2369104 | 38686817 | 38686866 | 1 | rs2869 | 38686854 | 0 | NA | NA | rs12147991 |
| TRIP10 | 19 | ILMN_2161746 | 6702277 | 6702326 | 2 | rs1049230-rs1049232 | 6702281-6702293 | 0 | NA | NA |  |
| TRPC4AP | 20 | ILMN_2402805 | 33054039 | 33054088 | 1 | rs1058003 | 33054078 | 0 | NA | NA | rs6088692-rs6120816-rs1885119 |
| TSEN34 | 19 | ILMN_1673111 | 59389022-59389030 | 59389029-59389071 | 1 | NA-rs2289145 | NA-59389058 | 0 | NA | NA |  |
| TSEN34 | 19 | ILMN_2368292 | 59389241 | 59389290 | 1 | rs4042 | 59389274 | 0 | NA | NA |  |
| TUBB | 6 | ILMN_2101885 | 30800937 | 30800986 | 2 | rs8233-rs9500863 | 30800944-30800986 | 1 | rs8233 | 30800944 | NA/rs9500867-rs7749235-rs2270172-rs11757306-rs6928738-rs9262551- |
| UBE3C | 7 | ILMN_2181363 | 156754389 | 156754438 | 1 | rs7807 | 156754403 | 0 | NA | NA |  |
| UNC45A | 15 | ILMN_2395932 | 89298220 | 89298269 | 1 | rs11207 | 89298243 | 0 | NA | NA |  |
| UNC84B | 22 | ILMN_2099301 | 37460760 | 37460809 | 1 | rs17032 | 37460789 | 0 | NA | NA |  |
| UPF3B | X | ILMN_2397627 | 118852441 | 118852490 | 1 | rs2239962 | 118852474 | 0 | NA | NA |  |
| VAPA | 18 | ILMN_2405190 | 9949473 | 9949522 | 1 | rs3069 | 9949493 | 1 | rs3069 | 9949493 |  |
| VAV3 | 1 | ILMN_2290068 | 108101443-108104929 | 108101474-108104946 | 1 | rs17541972-NA | 108101448-NA | 0 | NA | NA |  |
| VAV3 | 1 | ILMN_2399463 | 107915363 | 107915412 | 2 | rs8458-rs8676 | 107915379-107915388 | 0 | NA | NA |  |
| VRK3 | 19 | ILMN_2348403 | 55176034-55183441 | 55176082-55183441 | 1 | rs16981617-NA | 55176046-NA | 0 | NA | NA |  |
| WARS2 | 1 | ILMN_2323979 | 119375583 | 119375632 | 1 | rs8928 | 119375597 | 0 | NA | NA | rs1325939 |
| WDR33 | 2 | ILMN_1670172 | 128238666 | 128238715 | 1 | rs17534123 | 128238677 | 1 | rs17534123 | 128238677 |  |
| WDR47 | 1 | ILMN_2100085 | 109314765 | 109314814 | 1 | rs11803800 | 109314797 | 0 | NA | NA | rs11102735 |
| XYLT1 | 16 | ILMN_1830462 | 17103924 | 17103973 | 1 | rs1045885 | 17103934 | 0 | NA | NA |  |
| ZADH2 | 18 | ILMN_1795063 | 71039664 | 71039713 | 1 | rs9240 | 71039675 | 0 | NA | NA |  |
| ZDHHC9 | X | ILMN_1803824 | 128766635 | 128766684 | 1 | rs1053486 | 128766639 | 0 | NA | NA |  |
| ZHX2 | 8 | ILMN_2184966 | 124055800 | 124055849 | 2 | rs7758-rs2833 | 124055803-124055830 | 0 | NA | NA | rs7844465-rs3802266 |
| ZNF124 | 1 | ILMN_2110084 | 245386097 | 245386146 | 1 | rs3767857 | 245386112 | 0 | NA | NA |  |
| ZNF589 | 3 | ILMN_1654819 | 48286217 | 48286266 | 1 | rs12638476 | 48286257 | 0 | NA | NA |  |
| ZNF74 | 22 | ILMN_2383871 | 19091879 | 19091928 | 1 | rs887023 | 19091899 | 1 | rs887023 | 19091899 |  |
