# Supplementary material for: Genetics and Beyond – The Transcriptome of Human Monocytes and Disease Susceptibility
Source: PLoS One. 2010 May 18;5(5):e10693. doi: 10.1371/journal.pone.0010693 (PMC2872668; doi:10.1371/journal.pone.0010693)
Supplement: Table S2 — Loci identified in GWAS of BMI and BP - associations of lead/tag SNPs with phenotypes and expressions, and of expressions with phenotype in GHS. (0.07 MB DOC) [file pone.0010693.s002.doc]

### Table S2. Loci identified in GWAS of BMI and BP – associations of lead/tag SNPs with phenotypes and expressions, and of expressions with phenotype in GHS

|  |  |  |  |  |  |  | Associations in GHS | | | |
| --- | --- | --- | --- | --- | --- | --- | --- | --- | --- | --- |
| Lead SNP in meta-GWAS | Phenotype | Chr | Position (Mb) | Genes in region | Tag SNP in Affy 6.0 with r^2^ > 0.8 | r^2^ between lead SNP and tag SNP | Association between tag SNP and phenotype (*P* value) | eQTL associated with tag SNP | Association between tag SNP and eQTL (*P-*value) | Association between eQTL and phenotype (*P*-value) |
| **GWAS of BMI^a^** | |  |  |  |  |  |  |  |  |  |
| rs3101336 | BMI | 1 | 72.52 | *NEGR1* | rs2815752 | 1.00 | 0.47 | none |  |  |
| rs10913469 | BMI | 1 | 176.18 | *SEC16B, RASAL2* | rs527248 | 0.88 | 0.89 | none |  |  |
| rs2867125 | BMI | 2 | 0.61 | *TEM18* | rs2947411 | 1.00 | 0.86 | none |  |  |
| rs7647305 | BMI | 3 | 187.32 | *SFRS10, ETV5, DGKG* | rs1516728 | 0.81 | 0.17 | none |  |  |
| rs6265 | BMI | 11 | 27.64 | *LGR4, LIN7C, BDNF* | rs6265 | 1.00 | 0.19 | none |  |  |
| rs7138803 | BMI | 12 | 48.53 | *BCDIN3D, FAIM2* | rs7138803 | 1.00 | 0.09 | none |  |  |
| rs8049439 | BMI | 16 | 28.75 | *SH2B1, ATP2A1* | rs8049439 | 1.00 | 0.71 | *CCDC101* | 6.95E-39 | 0.0004 |
|  |  |  |  |  |  |  |  | *SPNS1* | 6.55E-64 | 0.0839 |
|  |  |  |  |  |  |  |  | *TUFM* | 3.11E-76 | 0.2609 |
| rs8050136 | BMI | 16 | 52.37 | *RPGRIP1L, FTO* | rs8050136 | 1.00 | 0.0034 | none |  |  |
| rs12970134 | BMI | 18 | 56.04 | *MC4R* | rs12970134 | 1.00 | 0.0777 | none |  |  |
| rs29941 | BMI | 19 | 39 | *CHST8, KCTD15* | rs29942 | 1.00 | 0.87 | *KCTD15* | 4.58E-15 | 0.0030 |
|  |  |  |  |  |  |  |  |  |  |  |
| **GWAS of BP^b^** | | |  |  |  |  |  |  |  |  |
| rs17367504 | SBP | 1 | 11.8 | *MTHFR* | rs7537765 | 0.94 | 0.17 | *MTHFR* | 1.14E-13 | 0.0281 |
| rs1918974 | DBP | 3 | 170.65 | *MDS1* | rs9290366 | 1.00 | 0.0027 | none |  |  |
| rs16998073 | DBP | 4 | 81.54 | *FGF5* | none |  |  |  |  |  |
| rs1530440 | DBP | 10 | 63.19 | *TMEM26* | rs1530440 | 1.00 | 0.45 | none |  |  |
| rs11191548 | SBP | 10 | 104.84 | *CNNM2/NT5C2* | rs12413409 | 1.00 | 0.0633 | *TMEM180* | 1.14E-10 | 0.9439 |
|  |  |  |  |  |  |  |  | *USMG5* | 1.23E-52 | 0.1069 |
| rs653178 | DBP | 12 | 110.47 | *ATXN2* | rs653178 | 1.00 | 0.0445 | none |  |  |
| rs1378942 | DBP | 15 | 72.86 | *CSK* | rs1378942 | 1.00 | 0.41 | *ULK3* | 3.21E-35 | 0.3810 |
| rs12946454 | SBP | 17 | 40.56 | *PLCD3* | rs11657325 | 0.88 | 0.0793 | *DCAKD* | 1.30E-12 | 0.4629 |
| rs16948048 | DBP | 17 | 44.79 | *ZNF652* | rs16948048 | 1.00 | 0.0204 | *GNGT2* | 2.63E-10 | 0.8040 |
|  |  |  |  |  |  |  |  |  |  |  |

*^a^ Data from Thorleifsson et al., Nature Genet 2009, 41:18-24; ^b^ Data from Newton-Cheh et al., Nature Genet 2009, 41:666-76; For several loci, we observed a significant association between the lead/tag SNP and a cis eQTL despite no correlation between phenotype and expression. In these cases, the best eSNP associated with the eQTL was also different from the lead/tag SNP associated with the phenotype and the r^2^ between the two SNPs was low. Such cases reflect a situation where two distinct loci are co-localized by chance in the same genomic region, one being responsible for the phenotype variability and the other one for the expression variability. The LD between SNPs in the region explains that most of them are co-associated by chance with the phenotype and the expression trait.*
