# Supplementary material for: Genetics and Beyond – The Transcriptome of Human Monocytes and Disease Susceptibility
Source: PLoS One. 2010 May 18;5(5):e10693. doi: 10.1371/journal.pone.0010693 (PMC2872668; doi:10.1371/journal.pone.0010693)
Supplement: Table S3 — Sets of gene expressions robustly and independently associated with each risk factor in the validation samples. (0.21 MB DOC) [file pone.0010693.s003.doc]

### Table S3. Sets of gene expressions robustly and independently associated with each risk factor in the validation samples

| Gene expression | Risk factor | Statistics in the validation sample calculated over 250 replicates | | | | |
| --- | --- | --- | --- | --- | --- | --- |
|  |  | % of positive replicati-ons | Median t-test | Median *P*-value | Minimum *P*-value | Maximum *P*-value |
| JARID1D | GENDER | 0.99 | -5.02 | 6.5E-07 | 6.6E-21 | 1.9E-02 |
| RPS4Y1 | GENDER | 0.99 | -4.65 | 3.9E-06 | 3.2E-16 | 3.8E-03 |
| RPS4Y2 | GENDER | 0.97 | -5.10 | 4.3E-07 | 2.1E-16 | 1.9E-03 |
| EIF1AX | GENDER | 0.95 | 5.54 | 4.3E-08 | 3.1E-16 | 2.3E-02 |
| EIF1AY | GENDER | 0.94 | -4.68 | 3.5E-06 | 4.3E-16 | 5.4E-02 |
| UTY | GENDER | 0.91 | -4.11 | 4.4E-05 | 3.8E-09 | 7.7E-02 |
| ZFX | GENDER | 0.81 | 3.28 | 1.1E-03 | 1.7E-07 | 2.4E-01 |
| ARSD | GENDER | 0.76 | 5.07 | 5.2E-07 | 7.1E-18 | 9.4E-03 |
| XIST | GENDER | 0.74 | 3.39 | 7.3E-04 | 2.8E-13 | 9.9E-01 |
| PRKX | GENDER | 0.56 | 2.68 | 7.6E-03 | 3.5E-06 | 3.7E-01 |
| TMSB4Y | GENDER | 0.56 | -2.50 | 1.3E-02 | 1.8E-05 | 6.5E-01 |
| ZFY | GENDER | 0.50 | -2.45 | 1.4E-02 | 1.0E-07 | 7.7E-01 |
| HDHD1A | GENDER | 0.50 | 2.45 | 1.5E-02 | 7.1E-07 | 5.0E-01 |
| CCDC106 | GENDER_AUTOSOM. | 1.00 | -7.50 | 1.9E-13 | 5.9E-26 | 9.0E-06 |
| MMEL1 | GENDER_AUTOSOM. | 0.99 | -5.39 | 9.4E-08 | 1.9E-15 | 4.1E-03 |
| ANKRD57 | GENDER_AUTOSOM. | 0.98 | 6.26 | 6.7E-10 | 1.2E-18 | 9.6E-03 |
| CXCR7 | GENDER_AUTOSOM. | 0.98 | -5.91 | 5.4E-09 | 7.4E-19 | 3.8E-03 |
| FCGR2B | GENDER_AUTOSOM. | 0.97 | 5.60 | 3.1E-08 | 3.5E-18 | 6.3E-04 |
| SOX15 | GENDER_AUTOSOM. | 0.97 | 4.72 | 2.8E-06 | 7.1E-11 | 1.1E-02 |
| FCGBP | GENDER_AUTOSOM. | 0.96 | 4.98 | 7.8E-07 | 1.2E-13 | 3.7E-02 |
| LPXN | GENDER_AUTOSOM. | 0.95 | -5.28 | 1.7E-07 | 2.8E-16 | 1.4E-02 |
| CD24 | GENDER_AUTOSOM. | 0.91 | -4.50 | 8.0E-06 | 1.7E-11 | 4.4E-02 |
| HOXA9 | GENDER_AUTOSOM. | 0.86 | 4.58 | 5.5E-06 | 2.3E-10 | 1.8E-02 |
| PTK6 | GENDER_AUTOSOM. | 0.86 | 4.24 | 2.6E-05 | 5.7E-13 | 3.8E-02 |
| DDX43 | GENDER_AUTOSOM. | 0.72 | -3.42 | 6.6E-04 | 1.6E-07 | 1.4E-01 |
| RAB11FIP1 | GENDER_AUTOSOM. | 0.61 | 4.71 | 2.9E-06 | 4.9E-16 | 1.7E-01 |
| PTK2 | GENDER_AUTOSOM. | 0.54 | 4.31 | 1.8E-05 | 7.7E-13 | 5.1E-01 |
| CLEC4G | GENDER_AUTOSOM. | 0.52 | -5.06 | 5.4E-07 | 3.7E-11 | 4.8E-03 |
| ADARB1 | GENDER_AUTOSOM. | 0.46 | 3.85 | 1.3E-04 | 1.3E-09 | 7.9E-01 |
| PROK2 | GENDER_AUTOSOM. | 0.45 | 3.08 | 2.1E-03 | 2.0E-08 | 2.7E-01 |
| MYBPH | GENDER_AUTOSOM. | 0.44 | 3.07 | 2.2E-03 | 1.2E-06 | 9.6E-01 |
| PER3 | GENDER_AUTOSOM. | 0.42 | -2.34 | 2.0E-02 | 2.0E-05 | 6.8E-01 |
| TPPP3 | GENDER_AUTOSOM. | 0.40 | 3.33 | 9.1E-04 | 2.4E-11 | 3.3E-01 |
| MPO | GENDER_AUTOSOM. | 0.38 | -2.43 | 1.5E-02 | 2.9E-06 | 9.2E-01 |
| FAM24B | GENDER_AUTOSOM. | 0.38 | -3.25 | 1.2E-03 | 6.0E-08 | 3.3E-01 |
| EMR3^$^ | GENDER_AUTOSOM. | 0.37 | 2.95 | 3.3E-03 | 3.9E-07 | 8.3E-01 |
| ENOSF1 | GENDER_AUTOSOM. | 0.36 | -2.27 | 2.3E-02 | 3.8E-06 | 4.0E-01 |
| TPM2 | GENDER_AUTOSOM. | 0.33 | 4.35 | 1.6E-05 | 1.7E-10 | 4.7E-01 |
| PTTG1IP | GENDER_AUTOSOM. | 0.33 | 2.91 | 3.8E-03 | 9.3E-06 | 3.4E-01 |
| CELSR3 | GENDER_AUTOSOM. | 0.30 | -2.16 | 3.1E-02 | 3.5E-04 | 9.9E-01 |
| CD1A | GENDER_AUTOSOM. | 0.29 | 2.89 | 4.0E-03 | 1.4E-06 | 4.6E-01 |
| FOLR2 | GENDER_AUTOSOM. | 0.27 | 2.68 | 7.6E-03 | 8.8E-07 | 2.5E-01 |
| BOLA3 | GENDER_AUTOSOM. | 0.27 | -3.39 | 7.5E-04 | 4.0E-09 | 4.9E-01 |
| OPLAH | GENDER_AUTOSOM. | 0.25 | 2.98 | 3.0E-03 | 6.7E-08 | 4.9E-01 |
| PARP3 | AGE | 0.96 | 5.56 | 3.80E-08 | 1.00E-17 | 4.30E-03 |
| PDGFRB | AGE | 0.89 | 5.46 | 6.40E-08 | 1.10E-15 | 2.50E-02 |
| NEFH | AGE | 0.76 | -5.26 | 1.90E-07 | 2.20E-13 | 5.30E-04 |
| P2RY2 | AGE | 0.67 | -4.37 | 1.40E-05 | 1.50E-11 | 5.90E-02 |
| SPINK2 | AGE | 0.67 | -3.84 | 1.30E-04 | 9.30E-10 | 2.70E-01 |
| GPER | AGE | 0.59 | 3.55 | 4.10E-04 | 3.30E-08 | 1.20E-01 |
| NFKBIZ | AGE | 0.38 | 4.02 | 6.30E-05 | 3.80E-12 | 1.40E-01 |
| ZSCAN18 | AGE | 0.34 | -2.97 | 3.00E-03 | 1.10E-07 | 5.20E-01 |
| IGLL1 | AGE | 0.31 | -3.85 | 1.30E-04 | 1.70E-09 | 1.30E-01 |
| BLK | AGE | 0.31 | -3.21 | 1.40E-03 | 1.30E-07 | 2.80E-01 |
| ITM2C | AGE | 0.28 | -4.58 | 5.60E-06 | 1.50E-11 | 3.10E-01 |
| C1RL | AGE | 0.28 | 3.16 | 1.70E-03 | 2.60E-07 | 1.90E-01 |
| CX3CR1 | BMI | 0.91 | 5.35 | 1.20E-07 | 2.70E-19 | 4.30E-03 |
| MAP3K6 | BMI | 0.9 | 4.56 | 6.00E-06 | 1.10E-14 | 2.30E-02 |
| FCGBP | BMI | 0.77 | -4.33 | 1.70E-05 | 3.80E-11 | 1.00E-01 |
| CD209 | BMI | 0.56 | -3.94 | 8.90E-05 | 4.80E-12 | 1.60E-01 |
| LYPD2 | BMI | 0.52 | 4.18 | 3.20E-05 | 4.00E-12 | 2.60E-02 |
| VSIG4 | BMI | 0.43 | -3.2 | 1.40E-03 | 3.00E-07 | 2.60E-01 |
| RPGRIP1 | BMI | 0.41 | -3.05 | 2.40E-03 | 2.00E-06 | 3.10E-01 |
| PACAP | BMI | 0.39 | -4.04 | 5.90E-05 | 3.00E-10 | 1.30E-01 |
| LGALS3BP | BMI | 0.38 | -3.98 | 7.60E-05 | 1.20E-09 | 1.80E-02 |
| ELA2 | BMI | 0.36 | 3.56 | 4.00E-04 | 7.70E-07 | 5.80E-02 |
| CD36 | BMI | 0.3 | 3.27 | 1.10E-03 | 2.60E-07 | 3.90E-01 |
| ABCA1 | BMI | 0.3 | -3.08 | 2.20E-03 | 2.50E-07 | 4.20E-01 |
| CRIP1 | SBP | 0.93 | 6.1 | 1.70E-09 | 4.40E-18 | 7.00E-03 |
| GFOD1 | SBP | 0.84 | 5.1 | 4.30E-07 | 5.70E-15 | 3.00E-02 |
| DHRS9 | SBP | 0.51 | -3.5 | 5.00E-04 | 3.20E-09 | 3.70E-01 |
| NR4A2 | SBP | 0.43 | 3.59 | 3.60E-04 | 2.50E-08 | 1.30E-01 |
| TSC22D3 | SBP | 0.39 | 3.3 | 1.00E-03 | 3.00E-08 | 4.90E-01 |
| ARID5B | SBP | 0.38 | 5.48 | 5.80E-08 | 5.70E-14 | 2.30E-03 |
| PAPSS2 | SBP | 0.37 | 2.5 | 1.30E-02 | 1.40E-08 | 4.90E-01 |
| HVCN1 | SBP | 0.28 | -3.14 | 1.80E-03 | 2.20E-07 | 5.80E-01 |
| GFOD1 | DBP | 0.77 | 4.75 | 2.50E-06 | 8.60E-10 | 1.20E-02 |
| CRIP1 | DBP | 0.42 | 3.69 | 2.50E-04 | 4.20E-10 | 6.70E-02 |
| TPPP3 | DBP | 0.35 | 3.44 | 6.20E-04 | 3.90E-11 | 9.80E-01 |
| NR4A2 | DBP | 0.34 | 2.86 | 4.30E-03 | 2.90E-05 | 2.90E-01 |
| EMP1 | DBP | 0.3 | 2.83 | 4.80E-03 | 5.70E-06 | 4.50E-01 |
| PRDM1 | HDL | 0.7 | -5.12 | 4.00E-07 | 2.50E-13 | 5.20E-01 |
| SCD | HDL | 0.65 | -4.87 | 1.40E-06 | 1.80E-11 | 4.20E-02 |
| DPEP2 | HDL | 0.37 | 3.75 | 1.90E-04 | 8.20E-11 | 1.00E-01 |
| TMEM43 | HDL | 0.34 | 3.2 | 1.50E-03 | 1.10E-08 | 3.20E-01 |
| BYSL | LDL | 0.34 | -3.22 | 1.30E-03 | 9.70E-06 | 1.10E-01 |
| ABCA1 | LDL | 0.29 | 2.78 | 5.70E-03 | 1.00E-04 | 3.60E-01 |
| MYLIP | TRIGLY | 0.72 | -4.26 | 2.30E-05 | 2.10E-13 | 1.60E-01 |
| PHGDH | TRIGLY | 0.48 | 3.33 | 9.20E-04 | 5.20E-07 | 4.10E-01 |
| ABCA1 | TRIGLY | 0.38 | -3.23 | 1.30E-03 | 5.00E-11 | 2.30E-01 |
| ELA2 | TRIGLY | 0.37 | 4.48 | 8.80E-06 | 2.80E-08 | 5.70E-02 |
| ABCG1 | TRIGLY | 0.31 | -3.71 | 2.30E-04 | 1.70E-08 | 8.20E-01 |
| SASH1 | TRIGLY | 0.3 | 3.61 | 3.30E-04 | 1.90E-06 | 1.30E-01 |
| FAM20A | CRP | 0.9 | 4.12 | 4.20E-05 | 4.40E-12 | 3.20E-02 |
| CETP | CRP | 0.82 | 4.76 | 2.30E-06 | 1.60E-12 | 3.30E-02 |
| FCGBP | CRP | 0.81 | -5 | 7.20E-07 | 1.90E-14 | 5.40E-03 |
| COL9A2 | CRP | 0.77 | -4.91 | 1.10E-06 | 4.90E-17 | 4.20E-02 |
| C1RL | CRP | 0.73 | 4.12 | 4.10E-05 | 1.00E-10 | 1.60E-01 |
| ADM | CRP | 0.62 | 4.79 | 2.00E-06 | 9.40E-17 | 1.30E-01 |
| CREB5 | CRP | 0.47 | 3.51 | 4.80E-04 | 1.40E-12 | 6.60E-01 |
| APBB1IP | CRP | 0.44 | 3.15 | 1.70E-03 | 4.10E-08 | 7.70E-01 |
| CX3CR1 | CRP | 0.38 | 4.06 | 5.50E-05 | 2.20E-09 | 5.80E-02 |
| C1QB | CRP | 0.38 | 3.12 | 1.90E-03 | 1.70E-06 | 2.00E-01 |
| MS4A4A | CRP | 0.37 | 3.05 | 2.30E-03 | 5.30E-09 | 2.20E-01 |
| FCER1A | CRP | 0.29 | -2.52 | 1.20E-02 | 8.20E-06 | 9.70E-01 |
| ALDH1A1 | CRP | 0.28 | -3.06 | 2.30E-03 | 2.80E-10 | 8.70E-01 |
| FLVCR2 | CRP | 0.25 | 2.52 | 1.20E-02 | 1.30E-07 | 9.30E-01 |
| SASH1 | SMOKING | 1 | 8.66 | 3.10E-17 | 2.50E-29 | 6.30E-09 |
| P2RY6 | SMOKING | 1 | 6.4 | 2.70E-10 | 1.00E-17 | 1.80E-05 |
| PTGDS | SMOKING | 1 | -5.88 | 6.30E-09 | 8.20E-16 | 1.10E-04 |
| PID1 | SMOKING | 0.91 | 4.84 | 1.60E-06 | 1.80E-13 | 8.30E-02 |
| CYP4F22 | SMOKING | 0.86 | -4.91 | 1.10E-06 | 5.00E-13 | 1.80E-01 |
| MMP25 | SMOKING | 0.84 | 4.64 | 4.20E-06 | 6.60E-14 | 2.00E-02 |
| WWC3 | SMOKING | 0.72 | 3.9 | 1.00E-04 | 1.40E-11 | 2.00E-01 |
| FUCA1 | SMOKING | 0.71 | 3.35 | 8.40E-04 | 1.80E-07 | 3.00E-01 |
| PDE4B | SMOKING | 0.7 | -3.61 | 3.30E-04 | 2.80E-08 | 4.80E-01 |
| STAB1 | SMOKING | 0.68 | 4.48 | 8.80E-06 | 5.20E-11 | 1.40E-01 |
| GFRA2 | SMOKING | 0.5 | 3.19 | 1.50E-03 | 7.00E-08 | 4.30E-01 |
| CLEC10A | SMOKING | 0.37 | 3.07 | 2.20E-03 | 8.00E-08 | 1.90E-01 |
| CAMK1D | SMOKING | 0.37 | -2.58 | 1.00E-02 | 6.40E-05 | 7.10E-01 |
| DHRS9 | SMOKING | 0.35 | -3.66 | 2.70E-04 | 3.80E-08 | 6.00E-01 |
| CNTNAP2 | SMOKING | 0.29 | 2.26 | 2.40E-02 | 4.50E-04 | 4.30E-01 |
| IQCK | SMOKING | 0.29 | -2.05 | 4.00E-02 | 1.30E-04 | 6.40E-01 |
| ITGB7 | SMOKING | 0.27 | 3.27 | 1.10E-03 | 6.90E-07 | 1.20E-01 |
| SMAD6 | SMOKING | 0.26 | 2.07 | 3.80E-02 | 2.10E-03 | 5.10E-01 |

*The screening/validation procedure was repeated 250 times. In each replicate, the association between the risk factor and the given expression trait was tested in the validation sample by multivariate regression analysis using a t-test. The statistics of the t-test and its corresponding P-value were calculated over the 250 replicates. The % of positive replications corresponds to the proportion of replicates in which the expression trait was significantly associated with the risk factor in the validation sample(P < 0.05).*

*GENDER_AUTOSOM. corresponds to the gender analysis restricted to autosomal genes.*

^$^ *EMR3* is the only gene in this list which is probed by a polymorphic probe (Table S1).
