# Supplementary material for: Genetics and Beyond – The Transcriptome of Human Monocytes and Disease Susceptibility
Source: PLoS One. 2010 May 18;5(5):e10693. doi: 10.1371/journal.pone.0010693 (PMC2872668; doi:10.1371/journal.pone.0010693)
Supplement: Methods S1 — GHS-Express database. (0.18 MB DOC) [file pone.0010693.s004.doc]

### GHS-Express database

<http://genecanvas.ecgene.net/uploads/ForReview/>

GHS-Express is a portable database using the SQLite SQL engine. The SQLite executable needs to be installed under LINUX, MAC or Windows. It can be downloaded at www.sqlite.org/download.html. The GHS_Express database is composed of tables that can be queried and connected using SQL queries. A set of SQL queries is provided as examples and can be used for several purposes by changing only a few parameters. To facilitate the use of the database, it is recommended to install a graphical “client” such as SQLiteman (sqliteman.com/index.php/page/4.html/) which is available for LINUX, MAC and Windows, or SQLiteExplorer ([www.singular.gr/sqlite/](http://www.singular.gr/sqlite/)) which is available for Windows only.

Once the SQLite client is installed, connecting to the GHS_Express database is simply done by opening it.


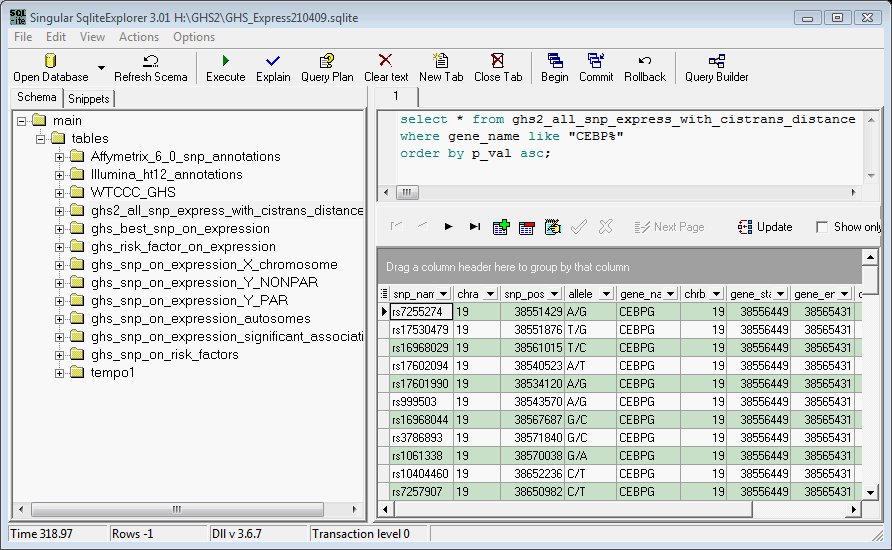


***GHS-Express tables***

**Affymetrix_6_0_snp_annotations** : Annotations for the Affymetrix 6.0 genotyping array

**Illumina_ht12_annotations** : annotations for the Illumina HT12 expression array

All tables starting with “ghs_” contain ghs summary data:

**ghs_all_snp_express_with_cistrans_distance** : All associations between SNPs and expressions with a p-value < 10^-5^ are reported (n=225615) together with the position of the SNP and of the associated gene. “cistransDistance” is 10^9^ when the SNP and gene are located on different chromosomes, 0 when the SNP is within the gene sequence, the number of base pairs separating the SNP from the gene sequence when the SNP and gene are located on the same chromosome, or -999 when the information is lacking.

**ghs_best_snp_on_expression** : This is a convenience table in which the best SNP for each gene was selected.

**ghs_risk_factors_on_expression**: All associations between SNPs and risk factors are shown. These associations are adjusted as explained in the paper. For example, the association between expression and BMI is adjusted on gender and age.

**ghs_snp_on_expression_autosomes**: Detailed associations between SNPs located on autosomes and expression.

**ghs_snp_on_expression_X_chromosome**: Associations between SNPs located on the X chromosome and expression in men and women separately and in both gender.

**ghs_snp_on_expression_Y_PAR** : Associations between SNPs on the pseudo-autosomal region (PAR) of chromosome Y and expression.

**ghs_snp_on_expression_Y_NONPAR**: Associations between SNPs on the non-pseudo-autosomal region (NONPAR) of chromosome Y and expression.

**ghs_snp_on_expression_significant_associations**: Summary table of significant associations between all SNPs and expression.

**ghs_snp_on_risk_factors** : Associations between SNPs and risk factors

***example of SQL query:***

*select * from ghs_snp_on_expression_autosomes*

*where genename like "CEBP%"*

*order by p_val asc;*

will retrieve all columns from the table **ghs_snp_on_expression_autosomes** for the gene symbols starting with CEBP and will order the results by increasing p value of association between SNPs and gene expressions.

**Example using R statistical language:**

R is a powerful statistical language that can be directly interfaced with the GHS_Express database.

For example the following script will load a SQL table in R for further statistical analyses

*## The necessary library is loaded and the connection with the database is established*

*ghs2dir <- "h://ghs2/"*

*library(RSQLite)*

*conn2 <- dbConnect("SQLite", dbname = paste(ghs2dir,"GHS_Express.sqlite",sep="") )*

*## a GHS_Express table is loaded*

*best_snp <- dbReadTable(conn2," ghs_best_snp_on_expression ")*

*## the table is now in the R environment and can be used for statistical analyses*
