# Supplementary material for: Genetics and Beyond – The Transcriptome of Human Monocytes and Disease Susceptibility
Source: PLoS One. 2010 May 18;5(5):e10693. doi: 10.1371/journal.pone.0010693 (PMC2872668; doi:10.1371/journal.pone.0010693)
Supplement: Methods S2 — Data quality checking. (0.21 MB DOC) [file pone.0010693.s005.doc]

### Data quality checking

**Checking for outliers and/or population stratification from GWV data.** The study population was examined for potential genetic heterogeneity using the first two principal components obtained by multidimensional scaling (MDS) of a matrix of pairwise IBS values between individuals. For this purpose, 18,000 SNPs were randomly selected from the 22 autosomal chromosomes and IBS was calculated using the PLINK software (Purcell et al. Am J Hum Genet 2007; 81: 559-575). MDS analysis was performed on the IBS distance matrix using the isoMDS function of the MASS R package. A first run of the analysis detected 17 subjects strongly deviating from the main cluster (Figure A). After exclusion of these 17 subjects, a second run led to the exclusion of 54 more subjects who deviated from the centre of the cluster by more than 2 SDs (Figure B). All these subjects were subsequently identified in sample records as of non-European ancestry. A third analysis on the remaining individuals showed no apparent stratification in the data (Figure C).

### Figure A. Checking for outliers and/or population stratification from GWV data – Run 1

The figure plots the coordonates of all subjects on the first 2 principal components otained by MDS analysis of a matrix of pairwise IBS values between subjects. After this first run, 17 outliers (red circles) were excluded.


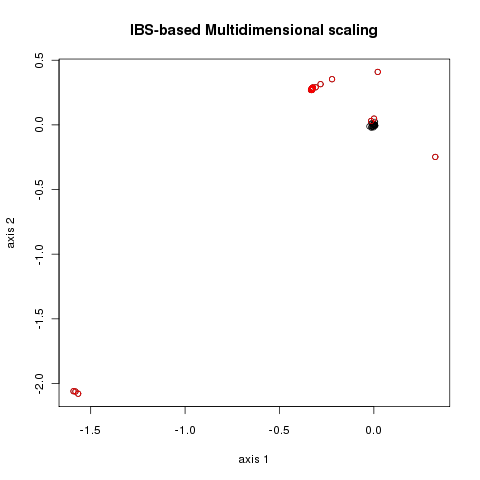


### Figure B. Checking for outliers and/or population stratification from GWV data – Run 2

A second run of the MDS analysis was performed after exclusion of the 17 outliers identified in run 1. After this second run, 54 additional subjects (red circles) were excluded.


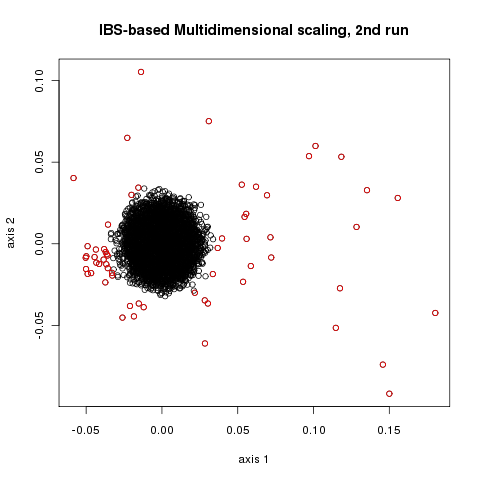


### Figure C. Checking for outliers and/or population stratification from GWV data – Run 3

The third run of MDS analysis shows that the remaining population is genetically homogeneous


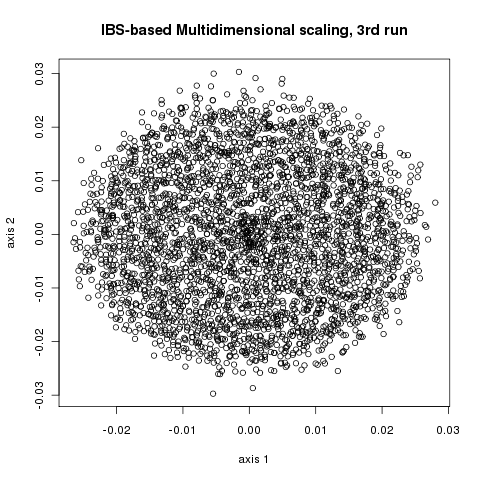


### Figure D. Replication of microarray GWE in 60 individuals


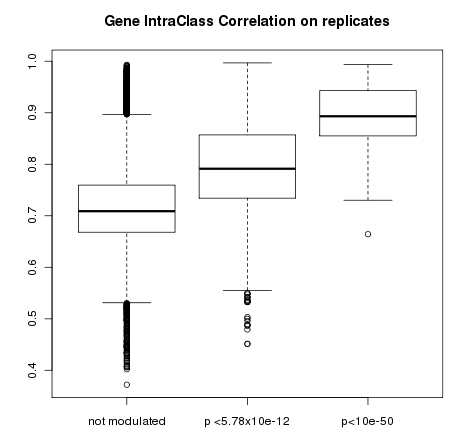


The figure displays box plots of the distribution of intra-individual correlations according to groups of genes: genes not genetically modulated; genes significantly modulated (*P* < 5.78x 10^-12^); genes highly significantly modulated (*P* < 10^-50^).

Duplicates and triplicates of micro-arrays were available in 66 and 10 individuals, respectively. Using these replicates, the intra-individual correlation of each gene expression was estimated by variance component analysis and the median corralation for all gene expressions was 0.7. The figure shows box plots of the distribution of intra-individual correlations in non-genetically modulated genes, all significantly modulated genes (*P* < 5.78 x 10^-12^) and the subset of highly modulated genes (*P* < 10^-50^). In these 3 categories of genes the median intra-individual correlation was 0.71, 0.79 and 0.89, respectively.

### Figure E. Checking for outliers in GWE data


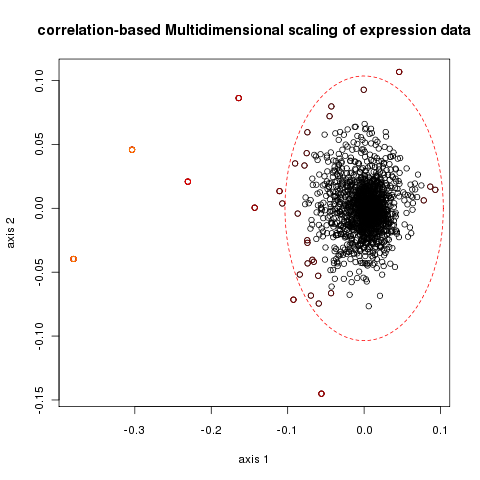


Similarly to GWV analysis, the first two components obtained by MDS were used to detect outliers in GWE data. The distance between any 2 individuals was computed as 1 minus the absolute correlation between the 2 arrays. Ten individuals whose data were located more than 4 s.d. away from the center of the main cluster were excluded from further analyses.
